# Extensive protein expression changes induced by pamidronate in RAW 264.7 cells as determined by IP-HPLC

**DOI:** 10.7717/peerj.9202

**Published:** 2020-05-21

**Authors:** Sang Shin Lee, Soung Min Kim, Yeon Sook Kim, Suk Keun Lee

**Affiliations:** 1 Department of Oral Pathology, College of Dentistry, Gangneung-Wonju National University, Gangneung, Gangwondo, South Korea; 2 Department of Oral and Maxillofacial Surgery, College of Dentistry, Seoul National University, Seoul, South Korea; 3 Department of Dental Hygiene, College of Health & Medical Sciences, Cheongju University, Cheongju, South Korea

**Keywords:** Pamidronate, Bisphosphonate, RAW 264.7 cells, Global protein expressions, Molecular signaling, IP-HPLC

## Abstract

**Background:**

Bisphosphonate therapy has become a popular treatment for osteoporosis, Paget’s disease, multiple myeloma, osteogenesis imperfecta, myocardial infarction, and cancer despite its serious side effects. Bisphosphonate-induced molecular signaling changes in cells are still not clearly elucidated.

**Methods:**

As bisphosphonates are primarily engulfed by macrophages, we treated RAW 264.7 cells (a murine macrophage cell line) with pamidronate and investigated global protein expressional changes in cells by immunoprecipitation high performance liquid chromatography (IP-HPLC) using 218 antisera.

**Results:**

Pamidronate upregulated proliferation-activating proteins associated with p53/Rb/E2F and Wnt/β-catenin pathways, but downregulated the downstream of RAS signaling, pAKT1/2/3, ERK-1, and p-ERK-1, and subsequently suppressed cMyc/MAX/MAD network. However, in situ proliferation index of pamidronate-treated RAW264.7 cells was slightly increased by 3.2% vs. non-treated controls. Pamidronate-treated cells showed increase in the expressions of histone- and DNA methylation-related proteins but decrease of protein translation-related proteins. NFkB signaling was also suppressed as indicated by the down-regulations of p38 and p-p38 and the up-regulation of mTOR, while the protein expressions related to cellular protection, HSP-70, NRF2, JNK-1, and LC3 were upregulated. Consequently, pamidronate downregulated the protein expressions related to immediate inflammation,cellular differentiation, survival, angiogenesis, and osteoclastogenesis, but upregulated PARP-1 and FAS-mediated apoptosis proteins. These observations suggest pamidronate affects global protein expressions in RAW 264.7 cells by stimulating cellular proliferation, protection, and apoptosis but suppressing immediate inflammation, differentiation, osteoclastogenesis, and angiogenesis. Accordingly, pamidronate appears to affect macrophages in several ways eliciting not only its therapeutic effects but also atypical epigenetic modification, protein translation, RAS and NFkB signalings. Therefore, our observations suggest pamidronate-induced protein expressions are dynamic, and the affected proteins should be monitored by IP-HPLC to achieve the therapeutic goals during treatment.

## Introduction

Bone undergoes constant remodeling maintained by a balance between osteoblasts and osteoclasts. Bisphosphonates inhibit the digestion of bone by causing osteoclasts to undergo apoptosis (Ito et al., 2001) and impair osteoclasts’ ability to form a ruffled border (Sato et al., 1991), to adhere to the bone surface, and to synthesize protons necessary for bone resorption. Furthermore, bisphosphonates suppress osteoclast activity by decreasing osteoclast progenitor development and recruitment (Cecchini et al., 1987; Endo et al., 1993). These diphosphate analogs inhibit intermediate enzymes of mevalonate pathway and are used to treat osteoporosis and Paget’s disease (historically osteitis deformans) (Abelson, 2008). In osteoporosis and Paget’s disease, IV zoledronic acid is the first-choice treatment for Paget disease because of its efficacy and ease of administration (Wat, 2014). The choice of zoledronic acid as the initial agent for most patients with active Paget disease is consistent with both the 2014 clinical practice guidelines of the Endocrine Society and the 2019 Paget’s Association guidelines (Singer et al., 2014).

Bisphosphonates bind calcium and are readily deposited in bone. They also change bone ultrastructures, for example, they obliterate Haversian canals and deposit irregular and thick reversal lines (Acevedo et al., 2015; Carmagnola et al., 2013; Kim et al., 2017c; Lee, 2013). The common side effects of bisphosphonates include bone pain, low blood calcium levels, nausea, and dizziness. In addition, bisphosphonate-related osteonecrosis of the jaw (BRONJ) may develop in patients who have used bisphosphonates long term (Marx et al., 2005; Ruggiero et al., 2004). Total 37 BRONJ cases out of 1,014 patients using bisphosphonates for osteoporosis treatment showed 62.6% were associated with intravenous and 37.4% with oral application (Hansen et al., 2013). The incidence of BRONJ is known to be low among patients treated with oral bisphosphonates (Sarasquete et al., 2009). The estimated prevalence of oral BRONJ was 0.05–0.07%. And the average oral bisphosphonate treatment duration was 43.1 months (range, 5–120 months) (Hong et al., 2010). Among the 320 osteoporotic patients who underwent tooth extraction, 11 developed BRONJ, reflecting an incidence rate of 3.44%. And the incidence of BRONJ increased with age, was greater in the mandible than the maxilla, and was associated with a duration of administration of more than 3 years (Jeong et al., 2017; Marx et al., 2005; Ruggiero et al., 2004). The pathophysiology of BRONJ is currently unclear. BRONJ has been attributed to infection (Chirappapha et al., 2017; Choi et al., 2017; Park et al., 2009), bisphosphonate-related osteonecrosis (Guimaraes et al., 2013), quantitative reduction of the vascularization (Kun-Darbois et al., 2018), local immune dysfunction (Hoefert et al., 2016b), genetic predisposition like polymorphisms on CYP2C8 gene (Sarasquete et al., 2009), etc.

In addition, to the anti-osteoporotic effect of bisphosphonates, adjunctive bisphosphonate therapy appears to be effective at managing periodontitis (Akram et al., 2017), fibrous dysplasia (Majoor et al., 2017), and Gorham-Stout disease (Hammer et al., 2005; Kim et al., 2015). Therefore, it is believed bisphosphonates may have several systemic effects such as anti-inflammatory, anti-proliferative, and anti-angiogenesis effects (Kamel, Geronikaki & Abdou, 2012; Ohlrich et al., 2016; Ribatti et al., 2008; Ribatti et al., 2008). However, the biological effects of bisphosphonates in different cells have not been clearly elucidated at the molecular level.

Pamidronate (pamidronate disodium or pamidronate disodium pentahydrate) is a nitrogen-containing bisphosphonate and used to prevent bone loss due to steroid use like glucocorticoid-induced low bone mineral density in children (Jayasena, Atapattu & Lekamwasam, 2015) or to inhibit calcium release from bone by impairing osteoclast-mediated bone resorption (Miyazaki et al., 2011), pamidronate is frequently used to treat high calcium levels (Polyzos et al., 2011). In addition, it has also been used as an experimental treatment for osteogenesis imperfecta and been studied for the treatment of complex regional pain syndrome (Chevreau et al., 2017).

Immunoprecipitation high-performance liquid chromatography (IP-HPLC) had been used previously by several authors to detect organic compounds including peptides quantitatively, but the technique used was complicated and of limited applicability (Clarke et al., 1998; Luo et al., 2013). Recently, a new IP-HPLC protocol was developed to determine protein expression levels in different biological fluids, such as blood serum, urine, saliva (Kim & Lee, 2015), inflammatory exudates (Kim et al., 2017a, 2017b, 2018), and different protein extracts from cells (Kim et al., 2019; Yoon et al., 2018b), liver (Yoon et al., 2018a), and cancer tissues (Kim et al., 2017d). The IP-HPLC is comparable to enzyme-linked immunosorbent assay (ELISA). The former uses protein A/G agarose beads in buffer solution and UV spectroscopy to determine protein concentrations, whereas the latter uses fluorescence-conjugated antibodies fixed in plastic wells and fluoroscopy. Furthermore, multiple trials have shown that IP-HPLC can be used to rapidly determine multiple protein levels accurately (<±5% standard deviation) and reproducibly. In the previous study (Yoon et al., 2018b), 64 proteins were assessed by IP-HPLC 4–8 times repeatedly and their results showed low error range <±5% standard deviation (shown in the raw data sheets of Supplemental Dataset 5).

When pamidronate is injected into blood vessels, it immediately chelates Ca^++^ (Ebetino et al., 2011; Fernandez, Vega & Goeta, 2002) and is bound to serum albumin (90% of tiludronate) (Sansom, Necciari & Thiercelin, 1995), and subsequently recognized by macrophages, which suggests its various pharmacologic effects may be associated with the cellular functions of pamidronate-laden macrophages. Therefore, the present in vitro study was undertaken to investigate the effects of pamidronate on protein expressions in RAW 264.7 macrophages by IP-HPLC.

## Materials and Methods

### RAW264.7 cell culture with pamidronate treatment

RAW 264.7 cells, an immortalized murine macrophage cell line (ATCC, Manassas, VA, USA), were cultured as previously described (Yoon et al., 2018b). About 70% confluent RAW 264.7 cells grown on Petri dish surfaces were treated with 6.5 μm disodium pamidronate (similar to the therapeutic dose, 1.5 mg/kg) (Sigma, Chicago, IL, USA) for 12, 24, or 48 h; control cells were treated with 1 mL of normal saline. Cultured cells were harvested with protein lysis buffer (PRO-PREP™; iNtRON Biotechnology INC., Gyeonggi-do, South Korea) and immediately preserved at −70 °C until required.

### In situ proliferation index of RAW 264.7 cells after 24 h of pamidronate treatment

RAW 264.7 cell proliferations were directly observed on plastic surfaces of Petri dishes after treatment with pamidronate at 6.5 μm for 12, 24, or 48 h, and compared with non-treated controls. When cells formed clusters containing 20–30 cells after 24 h of pamidronate treatment, ten representative histological images (taken at areas photographed before pamidronate treatment) were obtained using an inverted microscope (DP-73; Olympus Co., Tokyo, Japan). Cell numbers were obtained using the iSolution Lite program (IMT i-Solution Inc., Vancouver, BC, Canada), proliferation indices were calculated by dividing increases in cell numbers after 24 h and 48 h of culture by initial cell numbers and compared between pamidronate treatment groups and non-treated controls.

### Immunoprecipitation high-performance liquid chromatography

Protein extracts (about 100 μg) were subjected to immunoprecipitation using a protein A/G agarose column (Amicogen, Gyeongsangnam-do, South Korea). Protein A/G agarose columns were separately pre-incubated with 1 μg of 218 different antisera (147 monoclonal antibodies and 71 polyclonal antibodies (36 antibodies were purified with affinity columns)) specific to target amino acid motifs (product companies were listed in Table 1); for proliferation-related proteins (*n* = 11), cMyc/MAX/MAD signaling proteins (*n* = 3(1)), p53/Rb/E2F signaling proteins (*n* = 4(2)), Wnt/β-catenin signaling proteins (*n* = 6), epigenetic modification-related proteins (*n* = 7), protein translation-related proteins (*n* = 5), growth factor-related proteins (*n* = 18), RAS signaling proteins (*n* = 22), NFkB signaling proteins (*n* = 12(6)), up-regulated inflammatory proteins (*n* = 17), down-regulated inflammatory proteins (*n* = 27(1)), p53-mediated apoptosis-related proteins (*n* = 15(2)), FAS-mediated apoptosis-related proteins (*n* = 5(3)), cell survival-related proteins (*n* = 5(11), protection-related proteins (*n* = 12(13)), differentiation-related proteins (*n* = 11(11)), oncogenesis-related proteins (*n* = 10(10)), angiogenesis-related proteins (*n* = 14(9)), osteogenesis-related proteins (*n* = 11(4)), and control housekeeping proteins (*n* = 3) (numbers in parenthesis indicate number of overlapping antibodies, Table 1).

**Table 1:** Antibodies used in the study.

Proteins	No.	Antibodies
Proliferation-related	11	Ki-67*, PCNA*, CDK4*, MPM2*, PLK4*, cyclin D2, p14*, p16*, p21*, p27*, lamin A/C
cMyc/MAX/MAD network	3 (1)	cMyc*, MAX*, MAD-1* (p27)
p53/Rb/E2F signaling	4 (2)	p53, Rb-1^#^, E2F-1*, MDM2 (p21, CDK4)
Wnt/β-catenin signaling	6	Wnt1*, β-catenin*, APC*, snail*, TCF-1*, E-cadherin
Epigenetic modification	7	histone H1*, DMAP1*, KDM4D^$^, HDAC-10^$^, MBD4*, DNMT1*, PCAF*
Protein translation	5	DOHH^!^, DHS^!^, elF5A-1^!^, elF5A-2^!^, eIF2AK3*
Growth factor	18	FGF-1*, FGF-2*, FGF-7*, CTGF, HGFα*, TGF-β1^#^, TGF-β2*, TGF-β3*, SMAD4*, PDGF-A*, IGF-1*, IGFIIR*, GH*, GHRH*, HER1*, HER2*, ERβ*, Met*
RAS signaling	22	NRAS^$^, KRAS^$^, HRAS, STAT3*, PI3K, pAKT1/2/3, RAF-B*, JAK2^$^, JNK-1*, ERK-1*, p-ERK-1^$^, Rab 1*, mTOR, PTEN, NF-1, AKAP, caveolin-1, AMPK^@^, SOS-1*, SOS-2*, PKC*, p-PKC^@^
NFkB signaling	12 (6)	NFkB*, IKK*, TNFα, GADD45*, GADD153*, MDR, p38*, p-p38*, PGC-1α, ATF6, NRF-2*, SRC-1* (pAKT1/2/3, PTEN, ERK1*, p-ERK*, AMPK, mTOR^@^)
Upregulated inflammatory proteins	17	CD3, CD4, NCAM (CD56), CD80 (B7-1), Pdcd-1/1 (CD279), IL-8, IL-12, MMP-3^$^, -9^$^, -12^$^, CXCR4*, cathepsin C, MCP-1, COX2*, lactoferrin, versican, kininogen
Downregulated inflammatory proteins	27 (1)	IL-1*, IL-6*, IL-10*, IL-28*, cathepsin G*, cathepsin K*, COX1, lysozyme*, M-CSF, MMP-1, -2, -10, CD20, CD28, PECAM-1 (CD31), CD34, CD40, ICAM-1 (CD54), CD68, CD99, VCAM-1 (CD106), LTA4H^&^, LL-37, α1- antitrypsin ^&^, β-defensin-1, β-defensin-2, β-defensin-3 (TNFα^@^)
p53-mediated apoptosis	15 (2)	PUMA*, NOXA*, BCL2*, BAX*, BAD*, BAK*, BID*, AIF*, APAF-1*, caspase 9*, c-caspase 9*, caspase 3*, c-caspase 3*, PARP-1*, c-PARP-1* (MDM2*, p53*)
FAS-mediated apoptosis	5 (3)	FASL*, FAS*, FADD*, FLIP*, caspase 8* (BID*, caspase 3*, c-caspase 3*)
Cell survival-related	5 (11)	TERT*, survivin^@^, SP-1^@^, SP-3^@^, FAK (pAKT1/2/3, PTEN, AMPK, BCL2, NRF2, ATF6, PGC-1α, PKC, p-PKC, p38, p-p38)
Protection-related	12 (13)	HSP-27*, HSP-70*, HSP-90*, TGase 2^$^, LC3, mucin 1, mucin 4, HO-1*, SOD-1*, GSTO1*, SVCT2^&^, NOS-1^$^ (PLC-β2, PI3K, PKC*, p-PKC*, FAK*, caveolin-1*, PGC-1α*, AMPK, JNK-1, PLC-β2, PI3K, ATF6, NRF2)
Differentiation-related	11 (11)	p63^$^, vimentin, α-actin, PTCH-1, CyRP, SHH, cystatin A, S-100. integrin α5, HCAM (CD44) (caveolin-1, SP-1, SP-3, PLC-β2, PI3K, PKC, p-PKC, FAK, AP1M1, ICAM-1 (CD54), NCAM (CD56), PECAM (CD31))
Oncogenesis-related	10 (10)	BRCA1^&^, BRCA2^&^, NF-1*, ATM*, CEA^$^, 14-3-3*, maspin*, DMBT1*, YAP, PIM1 (MBD4, BCL2, SP-1, PTEN^&^, mucin 1, mucin 4, survivin^@^, TERT*, pAKT1/2/3*, mTOR)
Angiogenesis-related	14 (9)	HIF-1α^&^, VEGF-A*, VEGF-C*, angiogenin^$^, LYVE-1*, CMG2^$^, vWF^$^, FLT-4^$^, ET-1*, PAI-1*, VEGFR2*, p-VEGFR2, plasminogen*, leptin* (CD31, MMP-2, MMP-10, FGF-1, FGF-2, PDGF-A, PECAM-1 (CD31), VCAM (CD106), COX1)
Osteogenesis-related	11 (4)	OPG*, RANKL*, BMP-2*, BMP-3*, BMP-4*, ALP*, osteocalcin*, osteopontin*, osteonectin*, RUNX2*, osterix* (HSP-90, cathepsin K, CTGF, TGF-β1)
Control housekeeping proteins	3	α-tubulin*, β-actin*, GAPDH*
Total	218 (73)	

**Notes:**

* Santa Cruz Biotechnology, USA.

# DAKO, Denmark.

$ Neomarkers, CA, USA.

@ ZYMED, CA, USA.

& Abcam, Cambridge, UK.

! Kindly donated from M.H. Park in NIH, USA (Park & Wolff, 2018), the number of antibodies overlapped; ( ).

AIF, apoptosis inducing factor; AKAP, A-kinase anchoring proteins; ALP, alkaline phosphatase; AMPK, AMP-activated protein kinase; pAKT, v-akt murine thymoma viral oncogene homolog; p-Akt1/2/3 phosphorylated, p-Akt, Thr 308; APAF-1, apoptotic protease-activating factor 1; APC, adenomatous polyposis coli; ATF6, activating transcription factor 6; ATM, ataxia telangiectasia caused by mutations, BAD; BCL2 associated death promoter, BAK; BCL2 antagonist/killer, BAX; BCL2 associated X; BCL-2, B-cell leukemia/lymphoma-2; BID, BH3 interacting-domain death agonist; BMP-2, bone morphogenesis protein 2; BRCA1, breast cancer type 1 susceptibility protein; c-caspase 3, cleaved-caspase 3; CD3, cluster of differentiation 3; CDK4, cyclin dependent kinase 4; CEA, carcinoembryonic antigen; CMG2, capillary morphogenesis protein 2; COX-1, cyclooxygenase-2; CTGF, connective tissue growth factor; CXCR4, C-X-C chemokine receptor type 4; CyRP-1, cystein rich protein; DHS, deoxyhypusine synthase; DMAP1, DNA methyltransferase 1 associated protein; DMBT1, deleted in malignant brain tumors 1; DNMT1, DNA 5-cytosine methyltransferase 1; DOHH, deoxyhypusine hydroxylase; E2F-1, transcription factor; eIF2AK3 (PERK), eukaryotic translation initiation factor 2 (protein kinase R (PKR)-like endoplasmic reticulum kinase); elF5A-1, eukaryotic translation initiation factor 5A-1; ERK-1, extracellular signal-regulated protein kinase 1; ERβ, estrogen receptor beta; ET-1, endothelin-1; FADD, FAS associated via death domain; FAK, focal adhesion kinase; FAS, CD95/Apo1; FASL, FAS ligand; FGF-1, fibroblast growth factor-1; FLIP, FLICE-like inhibitory protein; FLT-4, Fms-related tyrosine kinase 4; GADD45, growth arrest and DNA-damage-inducible 45; GAPDH, glyceraldehyde 3-phosphate dehydrogenase; GH, growth hormone; GHRH, growth hormone-releasing hormone; GSTO1, glutathione S-transferase ω 1; HCAM (CD44), homing cell adhesion molecule; HDAC-10, histone deacetylase 10; HER1, human epidermal growth factor receptor 1; HGF-α, hepatocyte growth factor α; HIF-1α, hypoxia inducible factor-1α; HO-1, heme oxygenase 1; HRAS, GTPase HRas; HSP-70, heat shock protein-70; ICAM (CD54), intercellular adhesion molecule 1; IGF-1, insulin-like growth factor 1; IGFIIR, insulin-like growth factor 2 receptor; IKK, ikappaB kinase; IL-1, interleukin-1; JNK-1, Jun N-terminal protein kinase; KDM4D, Lysine-specific demethylase 4D; KRAS, V-Ki-ras2 Kirsten rat sarcoma viral oncogene homolog; LC3, microtubule-associated protein 1A/1B-light chain 3; LTA4H, leukotriene A4 hydrolase; LYVE-1, lymphatic vessel endothelial hyaluronan receptor 1; MAD-1, mitotic arrest deficient 1; MAX, myc-associated factor X; MBD4, methyl-CpG-binding domain protein 4; MCP-1, monocyte chemotactic protein 1; M-CSF, macrophage colony-stimulating factor; MDM2, mouse double minute 2 homolog; MDR, multiple drug resistance; MMP-1, matrix metalloprotease-1; MPM2, mitotic protein monoclonal 2; mTOR, mammalian target of rapamycin; cMyc, V-myc myelocytomatosis viral oncogene homolog; NFkB, nuclear factor kappa-light-chain-enhancer of activated B cells; NCAM (CD56), neural cell adhesion molecule 1; NF-1, neurofibromin 1; NFkB, nuclear factor kappa-light-chain-enhancer of activated B cells; NOS-1, nitric oxide synthase 1; NOXA, Phorbol-12-myristate-13-acetate-induced protein 1; NRAS, neuroblastoma RAS Viral Oncogene homolog; NRF2, nuclear factor (erythroid-derived)-like 2; OPG, osteoprotegerin; PAI-1, plasminogen activator inhibitor-1; PARP-1, poly-ADP ribose polymerase 1; c-PARP-1, cleaved-PARP-1; PCNA, proliferating cell nuclear antigen; Pdcd-1/1 (CD279), programed cell death protein 1; PDGF-A, platelet-derived growth factor-A; PECAM-1 (CD31), platelet endothelial cell adhesion molecule-1; PGC-1α, peroxisome proliferator-activated receptor gamma coactivator 1-α; PI3K, phosphatidylinositol-3-kinase; PIM-1, Proto-oncogene serine/threonine-protein kinase 1; PKC, protein kinase C; PLC-β2, 1-phosphatidylinositol-4,5-bisphosphate phosphodiesterse β-2; PLK4, polo like kinase 4 or serine/threonine-protein kinase; PTEN, phosphatase and tensin homolog; PUMA, p53 upregulated modulator of apoptosis; Rab 1, Rab GTPases; RAF-B, v-Raf murine sarcoma viral oncogene homolog B; RANKL, receptor activator of nuclear factor kappa-B ligand; Rb-1, retinoblastoma-1; RUNX2, Runt-related transcription factor-2; SHH, sonic hedgehog; SMAD4, mothers against decapentaplegic, drosophila homolog 4; SOD-1, superoxide dismutase-1; SOS-1, son of sevenless homolog 1; SP-1, specificity protein 1; SRC1, steroid receptor coactivator-1; STAT3, signal transducer and activator of transcription-3; SVCT2, sodium-dependent vitamin C transporter 2; TERT, human telomerase reverse transcriptase; TGase-2, transglutaminase 2; TGF-β1, transforming growth factor-β1; TNFα, tumor necrosis factor-α; VCAM, vascular cell adhesion molecule-1; VEGF-A, vascular endothelial growth factor A; VEGFR2, vascular endothelial growth factor receptor 2; p-VEGFR2, vascular endothelial growth factor receptor 2 (Y951); vWF, von Willebrand factor; Wnt1, proto-oncogene protein Wnt-1; YAP, Yes-associated protein.

Briefly, each protein sample (about 100 μg) was mixed with 5 mL of binding buffer (150 mm NaCl, 10 mm Tris pH 7.4, 1 mm EDTA, 1 mm EGTA, 0.2 mm sodium vanadate, 0.2 mm PMSF, 0.5% NP-40, and mixture of protein inhibitors (Sigma, Chicago, IL, USA)) and incubated with protein A/G agarose beads (200 μl, AmicogenGyeongsangnam-do, South Korea) bound with objective antibody on a rotating stirrer for 1 h at 4 °C. After washing beads with PBS (phosphate buffered saline solution), target proteins were eluted using 150 μl of IgG elution buffer (Pierce, Appleton, WI, USA). Immunoprecipitated proteins were analyzed using a HPLC unit (1100 series; Agilent, Santa Clara, CA, USA) equipped with a reverse phase column and a micro-analytical detector system (SG Highteco, Seoul, South Korea), using 0.15 M NaCl/20% acetonitrile solution at 0.4 mL/min for 30 min, and proteins were detected using a UV spectrometer at 280 nm. Control and experimental samples were run sequentially to allow comparisons. For IP-HPLC, whole protein peak areas (mAU*s) were mathematically calculated with analytical algorithm (see Supplemental Data 1) by subtracting negative control antibody peak areas, and protein expression levels (mAU) were compared and normalized using the square roots of protein peak areas. Analyses were repeated two to six times to achieve mean standard deviations of ≤±5% (RAW data, Supplemental Data 2). Objective protein expression level (%) between experiment and control groups were calculated and results were analyzed using the standard error of the mean (Kim et al., 2019; Yoon et al., 2018a, 2018b).

The housekeeping proteins normal β-actin, α-tubulin, and glyceraldehyde 3-phosphate dehydrogenase (GAPDH) were also used as internal controls. Expressional changes of housekeeping proteins were adjusted to <±5% using a proportional basal line algorithm. Protein expressional changes of ≤±5%, ±5–10%, ±10–20%, and ≥±20% change were defined as minimal, slight, meaningful, or marked, respectively.

When the IP-HPLC results were compared with the western blot data of cytoplasmic housekeeping protein (β-actin), the former exhibiting minute error ranges less than ±5% and could be analyzed statistically, while the latter showed a large error range of more than 20%, and thus it was almost impossible to analyze them statistically (see Supplemental Data 3). Therefore, the present study utilized IP-HPLC to statically analyze global protein expression changes in pamidronate-treated RAW 264.7 cells rather than Western blot method (Seo et al., 2019).

### Statistical analysis

Proportional data (%) of experimental and control groups were plotted, and analyses were repeated two to six times until standard deviations were ≤±5%. Results were analyzed by measuring standard error (${\rm s} = \pm \sqrt {{{{\rm \sigma} ^2}}/n}$). The expressions of control housekeeping proteins, that is, β-actin, α-tubulin, and glyceraldehyde 3-phosphate dehydrogenase (GAPDH) non-responsive (≤5%) to 12, 24, or 48 h of pamidronate treatment.

## Results

### In situ proliferation index of RAW 264.7 cells after 24 h of pamidronate treatment

**Figure 1:**
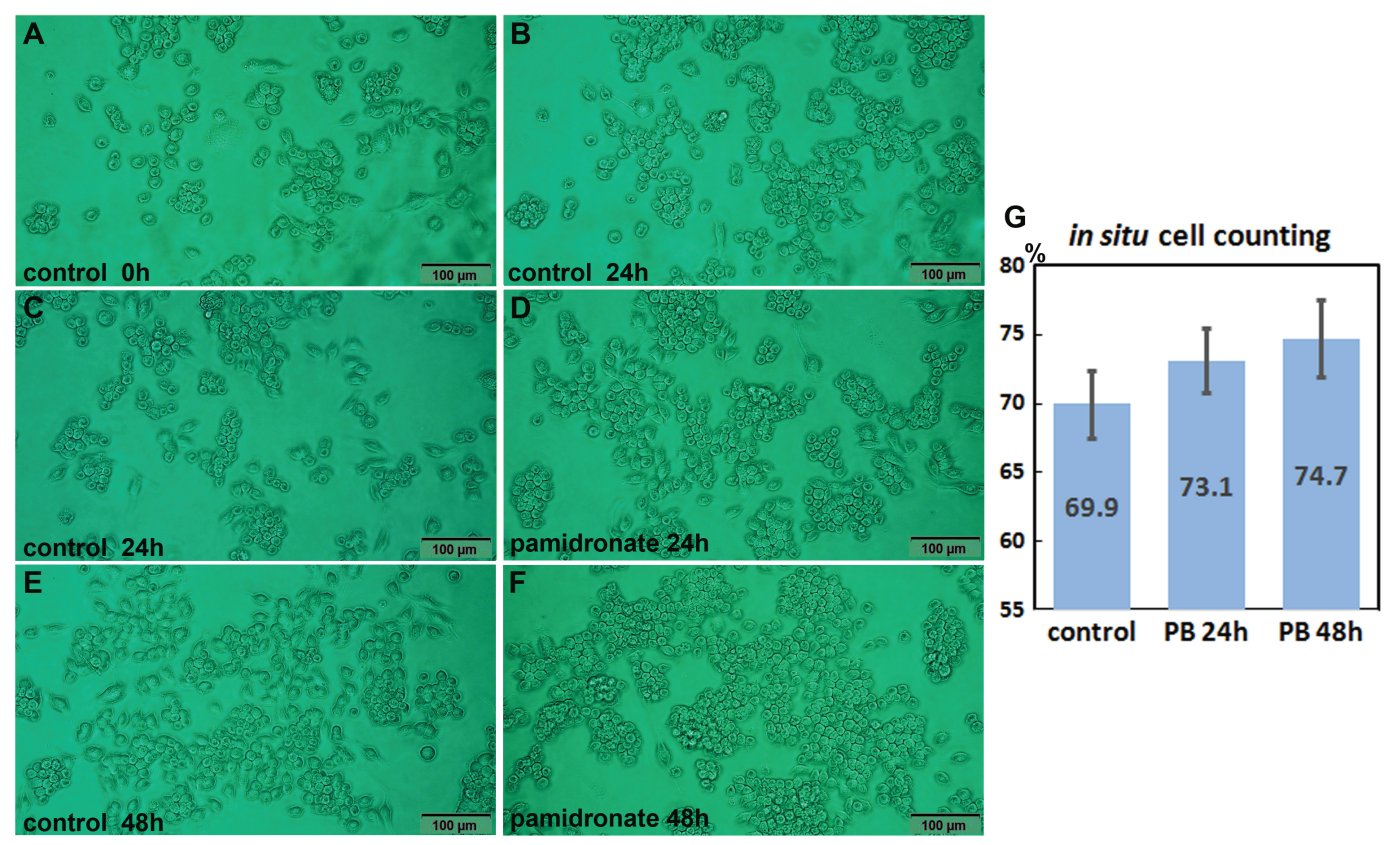
In situ proliferation assay of RAW 264.7 cells. Increases in cell numbers were determined by counting on Petri dishes (A–F), and proliferation indices (%) were calculated by expressing cell growths (final-initial cell counts) as percentages of initial cells counts. Pamidronate-treated (6.5 μm) RAW 264.7 cells had a slightly higher mean proliferation index (73.1 ± 2.32% at 24 h and 74.7 ± 2.8% at 48 h) than non-treated controls (69.9 ± 2.46%) (G) PB: pamidronate.

Both 6.5 μm pamidronate-treated RAW 264.7 cells and non-treated controls proliferated on Petri dishes and formed large cell clusters after 24 h of culture (Figs. 1A–1F). The in situ proliferation index of pamidronate-treated RAW 264.7 cells was 73.1 ± 2.32% at 24 h, 74.7 ± 2.8% at 48 h, and that of non-treated RAW 264.7 cells was 69.9 ± 2.46% by the in situ proliferation assay (Fig. 1G). These results indicate pamidronate slightly elevated mitosis of RAW 264.7 cells, murine macrophages, by 3.2% in 24 h and 4.8% in 48 h of culture.

### Effects of pamidronate on the expressions of proliferation-related proteins in RAW 264.7 cells

**Figure 2:**
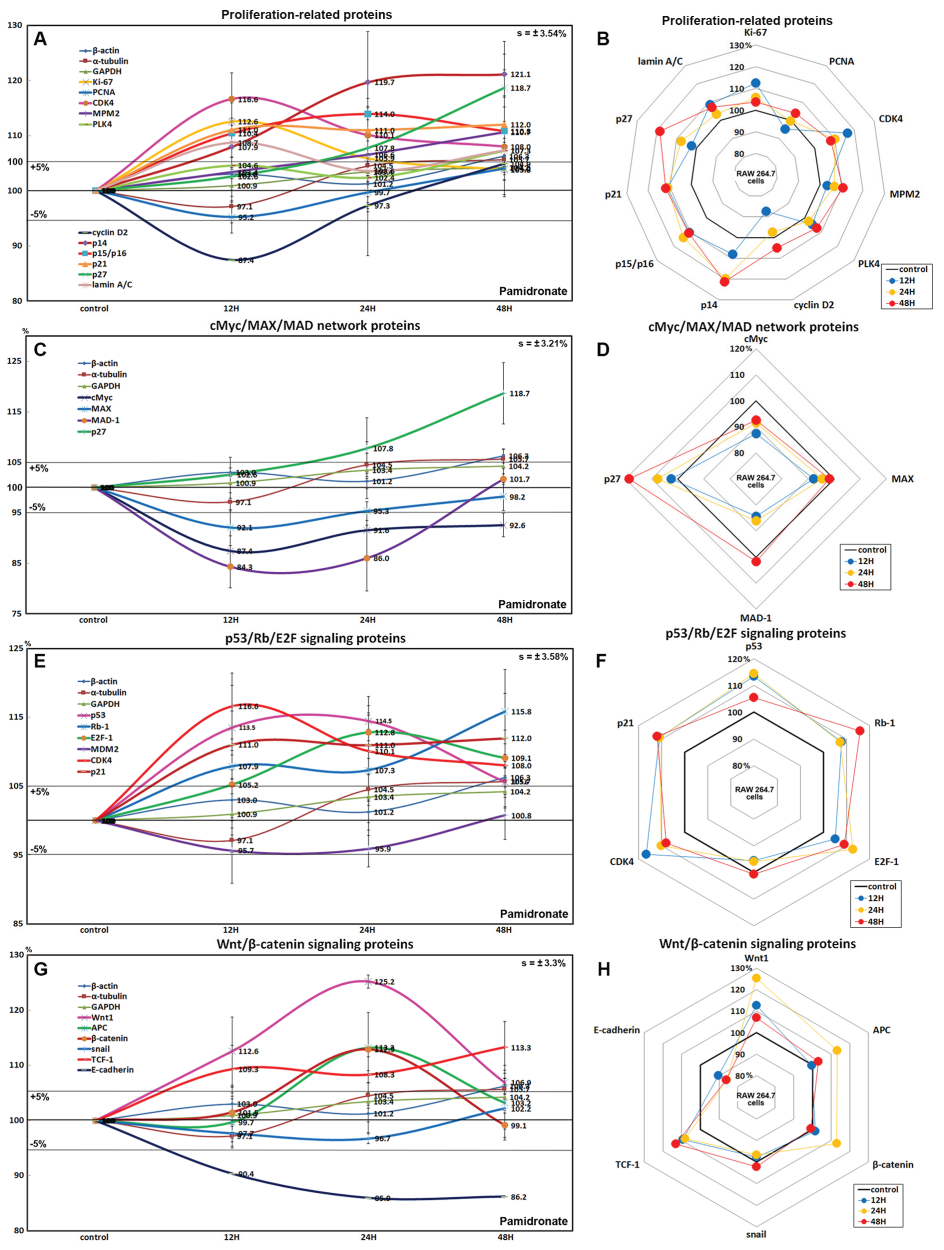
Expressions of proliferation-related proteins, cMyc/MAX/MAD network proteins, p53/Rb/E2F signaling proteins, and Wnt/β-catenin signaling proteins. Expressions of proliferation-related proteins (A and B), cMyc/MAX/MAD network proteins (C or D), p53/Rb/E2F signaling proteins (E and F), and Wnt/β-catenin signaling proteins (F or H) in pamidronate-treated RAW 264.7 cells as determined by IP-HPLC. Line graphs (A), (C), (E) and (G) show protein expressional changes on the same scale (%) vs. culture time (12, 24, or 48 h), whereas the star plots (B, D, F and H) show the differential expression levels of proteins after 12, 24, or 48 h of treatment on appropriate scales (%). Standard error (s).

RAW 264.7 cells treated with 6.5 μm pamidronate for 12, 24, or 48 h exhibited increases in levels of proliferation-activating proteins, Ki-67 (by 12.6%), proliferation cell nuclear antigen (PCNA, 4%), cyclin dependent kinase 4 (CDK4, 10.1%), mitosis phase promoting factor (MPF) recognized by a mitosis-specific monoclonal antibody (MPM-2, 10.7%), polo-like kinase 4 (PLK4, 7.3%), cyclin D2 (4.9%), and lamin A/C (8.7%) and also increases in proliferation-inhibiting proteins, p14 (21.1%), p15/16 (14%), p27 (18.7%) levels vs. non-treated controls. These expressional changes of proliferation-activating proteins became noticeable after 24 and 48 h of pamidronate treatment but remained at <±15%, but the proliferative activity of RAW 264.7 cells was limited by the increase of protein expressions of proliferation-inhibiting proteins (Figs. 2A and 2B). These results suggest pamidronate might have a mild proliferative effect on RAW 264.7 cells.

### Effects of pamidronate on the expressions of cMyc/MAX/MAD network proteins in RAW 264.7 cells

The expressions of cMyc and MAX decreased by 12.6% and 7.9%, respectively, after 12 h of pamidronate treatment and consistently decreased by 7.4% and 1.8%, respectively, at 48 h vs. non-treated controls, whereas MAD-1 expression decreased by a maximum of 15.7% after 12 h of treatment and slightly increased by 1.7% at 48 h. On the other hand, p27 expression increased by 18.7% after 48 h of treatment (Figs. 2C and 2D). These results indicate pamidronate suppressed cMyc/MAX/MAD network expressions and resulted low level of Myc-Max heterodimers which are strongly binding to E-box (CACGTG). These expressional changes of cMyc/MAX/MAD network proteins may negatively contribute to the proliferative effect of pamidronate on RAW 264.7 cells.

### Effects of pamidronate on the expressions of p53/Rb/E2F signaling proteins in RAW 264.7 cells

Pamidronate increased the expression of p53 in RAW 264.7 cells by 14.5% at 12 h but its increase was diminished by 8.7% at 48 h vs. non-treated controls, and decreased the expression of negative regulator of p53, MDM2, by 4.3% at 12 h. Rb-1 expression was also slightly increased by 7.9%, 7.3%, 15.8% at 12, 24, and 48 h, respectively. Notably, the expression of CDK4, activator of Rb-1 was increased by 16.6% at 12 h, although p21, CDK inhibitor was also increased by 11% at 12 h concurrent with the elevation of p53 expression. Resultantly, the expression of the objective transcription factor, E2F-1, increased by 12.8% at 24 h and by 9.1% at 48 h (Figs. 2E and 2F). This up-regulation of p53/Rb/E2F signaling by pamidronate may indicate the increase in the level of Rb-1 phosphorylation and positively affect RAW 264.7 cell proliferation.

### Effects of pamidronate on the expressions of Wnt/β-catenin signaling proteins in RAW 264.7 cells

The expressions of Wnt1, β-catenin, and adenomatous polyposis coli (APC) in RAW 264.7 cells were increased by 25.2%, 12.9%, and 8.7%, respectively, by pamidronate at 24 h vs. non-treated controls, while the expression of E-cadherin was reduced by 13.8% coincident with slight increase of snail expression by 2.2% at 48 h. Resultantly, the expression of the objective transcription factor T-cell factor 1 (TCF-1) was increased by 9.3% at 12 h and by 13.3% at 48 h (Figs. 2G and 2H). These findings regarding the up-regulation of Wnt/β-catenin signaling and downregulation of E-cadherin by pamidronate may have significantly increased RAW 264.7 proliferation.

### Effects of pamidronate on the expressions of epigenetic modification-related proteins in RAW 264.7 cells

**Figure 3:**
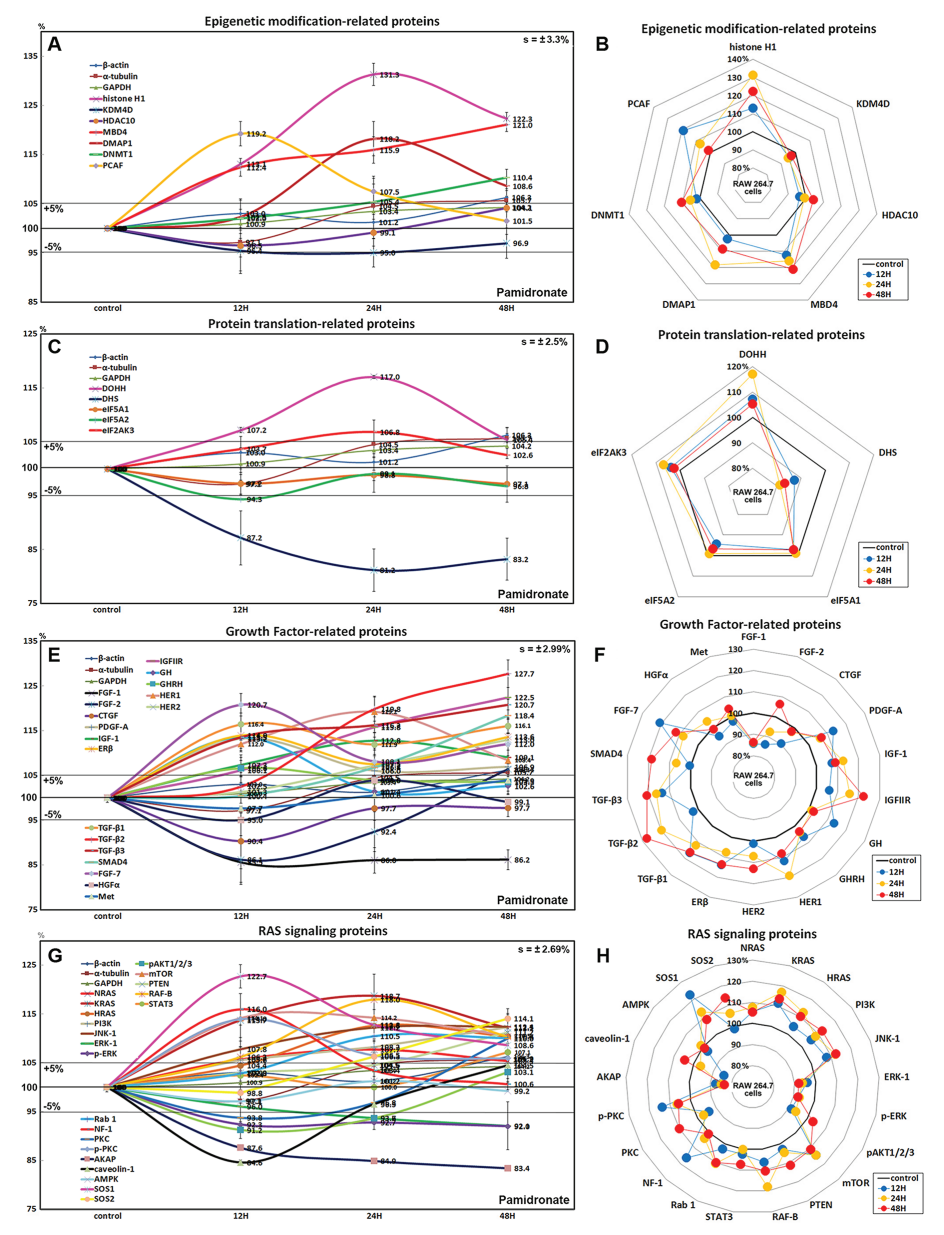
Expressions of epigenetic modification-related proteins, protein translation-related proteins, growth factors, and RAS signaling proteins. Expressions of epigenetic modification-related proteins (A and B), protein translation-related proteins (C or D), growth factors (E and F), and RAS signaling proteins (G or H) in pamidronate-treated RAW 264.7 cells as determined by IP-HPLC. Line graphs (A), (C), (E), and (G) show protein expressional changes on the same scale (%) vs. culture time (12, 24, or 48 h), whereas the star plots (B, D, F, and H) show the differential expression levels of proteins after 12, 24, or 48 h of treatment on appropriate scales (%). Standard error (s).

Histone H1 expression increased in pamidronate treated cells to 131.3% at 24 h and to 122.3% at 48 h vs. non-treated controls. Regarding histone modification, the expression of lysine-specific demethylase 4D (KDM4D) was 5% lower at 24 h, but that of histone deacetylase 10 (HDAC10) showed little change. With respect to DNA modification, DNA (cytosine-5)-methyltransferase 1 (DNMT1) expression was 10.4% higher at 48 h and those of DNA methyltransferase 1-associated protein 1 (DMAP1) and methyl-CpG binding domain 4 (MBD4) were 18.2% and 15.9% higher at 24 h, respectively, and were maintained at 8.6% and 21% higher at 48 h (Figs. 3A and 3B). These results suggest pamidronate increased histone and DNA methylation and subsequently hindered DNA transcription in RAW 264.7 cells, and that this epigenetic effect of pamidronate might be related to the down-regulation of various proteins.

### Effects of pamidronate on the expressions of translation-related proteins in RAW 264.7 cells

RAW 264.7 cells treated with pamidronate showed gradual reductions in protein translation-related protein levels vs. non-treated controls. Although deoxyhypusine hydroxylase (DOHH) expression slightly increased by 17% and 5.4% after 24 and 48 h of treatment, respectively, deoxyhypusine synthase (DHS) expression was consistently reduced by 18.8% and 16.8%, respectively, at these times. The protein expressions of objective factors of protein translation, that is, eukaryotic translation initiation factor 5A-1 (eIF5A-1) and eIF5A-2, were also reduced by 2.9% and 3.2% at 48 h, respectively, while that of eukaryotic translation initiation factor 2-α kinase 3 (eIF2AK3; an inactivator of eIF2) was increased by 6.8% at 24 h (Figs. 3C and 3D). We considered that the pamidronate-induced reductions in the expressions of translation-related proteins might cause global inactivation of cellular signaling. However, changes in the levels of these protein levels which are normally abundant in cells tended to remain at <±15% after 48 h of pamidronate treatment.

### Effects of pamidronate on the expressions of growth factor-related proteins in RAW 264.7 cells

RAW 264.7 cells treated with pamidronate for 48 h showed increases in the expressions of growth hormone (by GH, 13.5%), growth hormone-releasing hormone (GHRH, 6.6%), platelet-derived growth factor-A (PDGF-A, 13.2%), insulin-like growth factor-1 (IGF-1, 12.8%), IGF-2 receptor (IGFIIR, 22.5%), epidermal growth factor receptor (ErbB-1, HER1, 19.2%), HER2 (receptor tyrosine-protein kinase ErbB-2, 13%), transforming growth factor-β1 (TGF-β1, 16.4%), TGF-β2 (27.7%), TGF-β3 (20.7%), SMAD4 (18.4%), fibroblast growth factor-7 (FGF-7 known as a keratinocyte growth factor, 20.7%), and estrogen receptor β (ERβ, 14%) over 48 h vs. non-treated controls whereas the expressions of FGF-1, FGF-2, and CTGF decreased by 14%, 13.9%, and 9.6%, respectively. The expressions of other growth factor-related proteins, including those of hepatocyte growth factor α (HGFα) and Met, changed minimally (by ±5%) like the expressions of housekeeping proteins (Figs. 3E and 3F). These results indicate pamidronate influenced the expressions of many growth factors necessary for the growth and differentiation of RAW 264.7 cells, that is, it increases the expressions of GH, GHRH, PDGF-A, IGF-1, IGFIIR, HER1, HER2, TGF-β1, TGF-β2, TGF- β3, SMAD4, FGF-7, and ERβ, while reduces the expressions of extracellular matrix maturation, that is, FGF-1, FGF-2, and CTGF.

### Effects of pamidronate on the expressions of RAS signaling proteins in RAW 264.7 cells

Although many RAS upstream signaling proteins were upregulated by pamidronate, RAS downstream effector proteins were significantly downregulated. The increase in the expressions of KRAS (by 16.8%), NRAS (7.7%), HRAS (12.6%), phosphatidylinositol 3-kinase (PI3K, 12.3%), Jun N-terminal protein kinase-1 (JNK-1, 12.4%), mammalian target of rapamycin (mTOR, 14.2%), phosphatase and tensin homolog (PTEN, 11.2%), RAF-B (serine/threonine-protein kinase B-Raf, 18%), Rab 1 (GTPase, 10.5%), neurofibromin 1 (NF-1, 16%), protein kinase C (PKC, 10%), p-PKC (14%), son of sevenless homolog 1 (SOS-1, 22.7%), SOS-2 (14.1%), and signal transducer and activator of transcription-3 (STAT3, 7.2%) were found over 48 h of treatment vs. non-treated controls, while RAS downstream expressions of pAKT1/2/3, 5′ AMP-activated protein kinase (AMPK), extracellular signal–regulated kinase 1 (ERK-1), and p-ERK-1 were decreased by 8.8%, 2.9%, 7.9%, and 8%, respectively. And the expressions of A-kinase anchoring protein (AKAP) and caveolin-1 were also reduced by 16.6% and 15.4%, respectively (Figs. 3G and 3H). These results indicate pamidronate significantly reduced the expressions of the downstream effector proteins, ERK-1 and p-ERK-1, albeit many upstream proteins (KRAS, NRAS, HRAS, PI3K, JNK-1, mTOR, PTEN, RAF-B, Rab 1, NF-1, PKC, p-PKC, SOS-1, SOS-2, and STAT3), and thus, suggest RAS signaling (a major signal for cellular growth) was gradually attenuated in RAW 264.7 macrophages.

### Effects of pamidronate on the expressions of NFkB signaling proteins in RAW 264.7 cells

**Figure 4:**
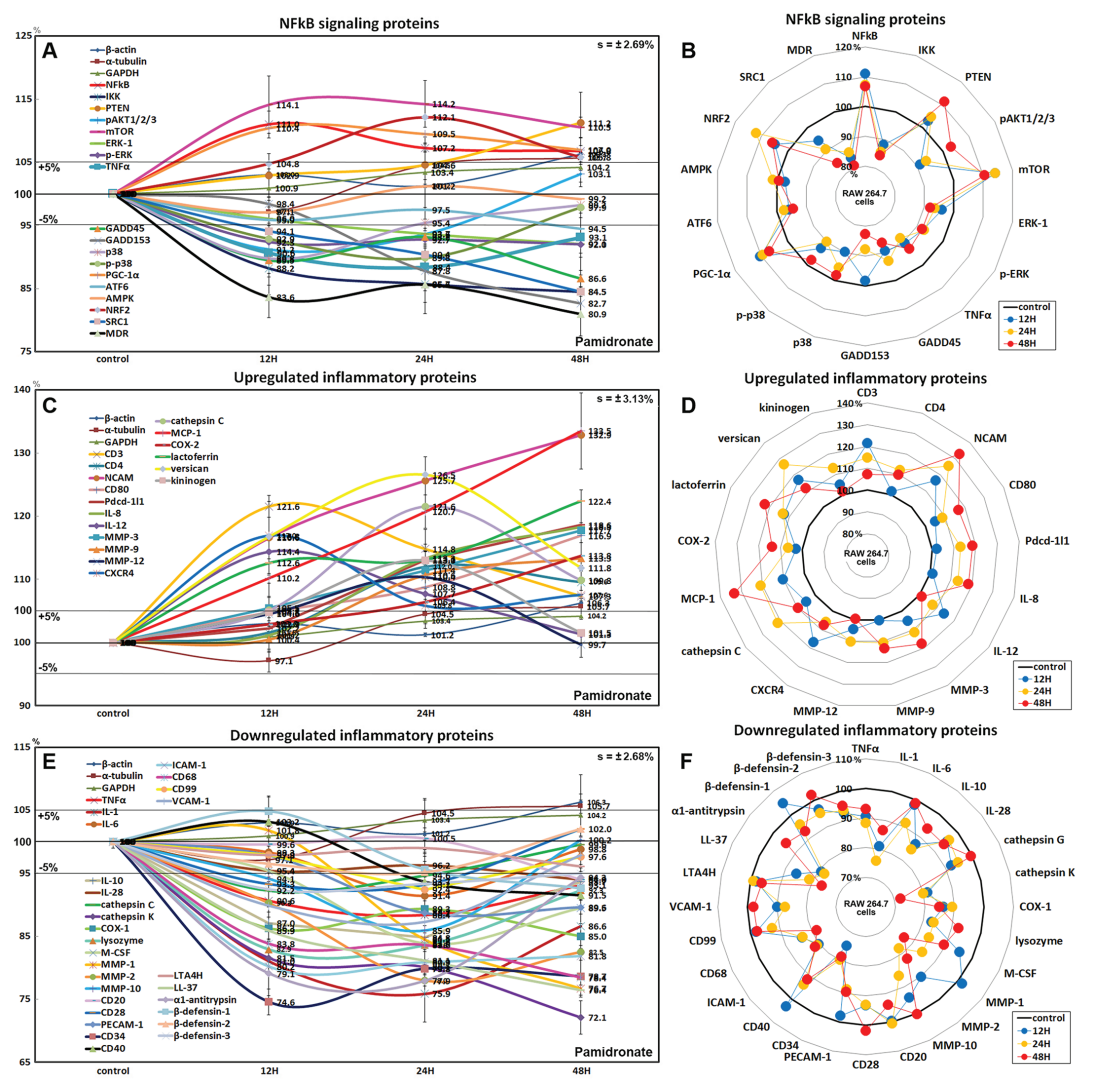
Expressions of NFkB signaling proteins, inflammatory proteins were upregulated, and inflammatory proteins downregulated. Expressions of NFkB signaling proteins (A and B), inflammatory proteins were upregulated (C or D), and inflammatory proteins downregulated (E and F) in pamidronate treated RAW 264.7 cells as determined by IP-HPLC. Line graphs (A), (C) and (E) show protein expressional changes on the same scale (%) vs. culture time (12, 24, or 48 h), whereas the star plots (B, D, and F) show the differential expression levels of proteins after 12, 24, or 48 h of treatment on appropriate scales (%). Standard error (s).

Pamidronate had different effects on the expressions of NFkB signaling proteins in RAW 264.7 cells. The expressions of NFkB upstream signaling proteins were increased by pamidronate, that is; nuclear factor kappa-light-chain-enhancer of activated B cells (NFkB, by 11%), PTEN (11.2%), mTOR (14.2%), peroxisome proliferator-activated receptor gamma coactivator 1-α (PGC-1α, 10.4%), and nuclear factor (erythroid-derived 2)-like 2 (NRF2, 12.1%), while the expressions of NFkB downstream effector proteins, pAKT1/2/3, growth arrest and DNA damage 45 (GADD45), GADD153, p38, p-p38, steroid receptor coactivator-1 (SRC1), and multi-drug resistance (MDR) were reduced by 8.8%, 13.4%, 17.3%, 10.2%, 10.2%, 15.5%, and 19.1%, respectively. The expression of ikappaB kinase (IKK) expressions was decreased by 15.5% after 48 h of treatment, and those of tumor necrosis factor α (TNFα) and activating transcription factor 6 (ATF6) only decreased by <5%. These results indicate the expressions of many proteins that enhance NFkB signaling tended to be downregulated by treatment with pamidronate for 48 h, that is, pAKT1/2/3, GADD45, GADD153, p38, p-p38, SRC1, MDR, and more, proteins suppressing NFkB signaling tended to be upregulated by pamidronate, that is, PTEN, mTOR, PGC-1α, and NRF2 (Figs. 4A and 4B). These results indicate pamidronate effectively suppressed NFkB signaling in RAW 264.7 cells.

### Effects of pamidronate on the expressions of upregulated inflammatory proteins in RAW 264.7 cells

The proteins upregulated by pamidronate were; CD3 (a T cell co-receptor constituting T cell receptor (TCR) complex, by 21.6%), CD4 (a co-receptor of the T cell receptor (TCR), 12%), neural cell adhesion molecule (NCAM, CD56, detecting natural killer cells, gamma delta (γδ) T cells, activated CD8+ T cells, and dendritic cells, 32.8%), CD80 found on the surface of dendritic cells, B cells, monocytes and antigen-presenting cells (16.9%), programmed cell death protein 1 (Pdcd-1/1, CD279, 18.9%), IL-8 (a chemoattractant cytokine, 18.3%), IL-12 (a T cell-stimulating factor, 11.4%), MMP-3 stromelysin-1 involved in wound repair, progression of atherosclerosis, and tumor initiation (17.7%), MMP-9 (a regulating factor for neutrophil migration, angiogenesis, and wound repair, 13.3%), MMP-12 (a macrophage metalloelastase contributing to elastin degradation, 10.4%), cathepsin C (a lysosomal exo-cysteine protease degrading various extracellular matrix components, 21.6%), C-X-C chemokine receptor type 4 (CXCR4, 17%), monocyte chemotactic protein-1 (MCP-1, an eotaxin, 33.5%), cyclooxygenase 2 (COX2, an important mediator of inflammation, 13.8%), versican (a large extracellular matrix proteoglycan that is involved with tissue homeostasis and inflammation, 26.5%), and kininogen (a constituent of the blood coagulation system as well as the kinin-kallikrein system, 13%) (Figs. 4C and 4D).

These results indicate pamidronate stimulated cell-mediated immunity and chronic inflammation by upregulation of CD3, CD4, NCAM, CD80, Pdcd-1/1, IL-8, IL-12, MMP-3, MMP-9, MMP-12, cathepsin C, CXCR4, MCP-1, COX2, versican, and kininogen in RAW 264.7 cells.

### Effects of pamidronate on the expressions of downregulated inflammatory proteins in RAW 264.7 cells

Proteins downregulated were tumor necrosis factor α (TNFα, by 11.6%), IL-1α (a “dual-function cytokine”, which means it plays a role in the nucleus by affecting transcription, apart from its extracellular receptor-mediated effects as a classical cytokine, 24.1%), IL-6 (an important mediator of fever and of the acute phase response, 8.6%), IL-10 (an anti-inflammatory cytokine, 15.2%), IL-28 which play a role in the adaptive immune response (6%), B-lymphocyte antigen CD20 (6%), CD28 which is necessary for T cell activation and survival (6.8%), PECAM-1 (CD31, a role for leukocyte transmigration, angiogenesis, and integrin activation, 11.3%), CD34 (a transmembrane phosphoglycoprotein protein which is expressed in early hematopoietic and vascular-associated tissues, 25.4%), CD40 (a costimulatory protein found on antigen presenting cells, 8.5%), intercellular adhesion molecule 1 (ICAM-1, CD54, 19.8%), CD68 (a marker for the various cells of the macrophage lineage, 21.6%), CD99 (MIC2, a heavily O-glycosylated transmembrane protein which is expressed in all leukocytes, 7.6%), vascular cell adhesion molecule-1 (VCAM, CD106, a role in leukocyte-endothelial cell signal transduction, 12.7%), cathepsin G (an important role in eliminating intracellular pathogens and breaking down tissues at inflammatory sites, 7.8%), cathepsin K (a lysosomal cysteine protease involved in bone remodeling and resorption, 27.9%), COX1 (prostaglandin G/H synthase 1 involved in cell signaling and maintaining tissue homeostasis, 15%), lysozyme (17.1%), macrophage colony-stimulating factor (M-CSF, 16.1%), MMP-1 (an interstitial collagenase, 23.3%), MMP-2 (a role for lymphangiogenesis, 22%), MMP-10 (stromelysin-2, 14.1%), leukotriene A4 hydrolase (LTA4H, 4.9%), cathelicidin antimicrobial peptides LL-37 (an antimicrobial peptide, 23.6%), α1-antitrypsin (a protease inhibitor, 22.1%), β-defensin 1 (a microbicidal and cytotoxic peptide, 7.4%), β-defensin 2 (a microbicidal and cytotoxic peptide, 4.8%), and β-defensin 3 (a microbicidal and cytotoxic peptide, 7.6%) over 48 h of pamidronate treatment (Figs. 4E and 4F).

These results indicate pamidronate inhibited innate immunity, immediate inflammatory rection, and wound repair processes by downregulation of TNFα, IL-1α, IL-6, IL-10, IL-28, CD20, CD28, PECAM-1, CD34, CD40, CD68, CD99, VCAM, cathepsin G, cathepsin K, COX1, lysozyme, M-CSF, MMP-1, MMP-2, MMP-10, LTA4H, LL-37, α1-antitrypsin, β-defensin 1, β-defensin 2, and β-defensin 3 in RAW 264.7 cells.

### Effects of pamidronate on the expressions of p53-mediated apoptosis-related proteins in RAW 264.7 cells

**Figure 5:**
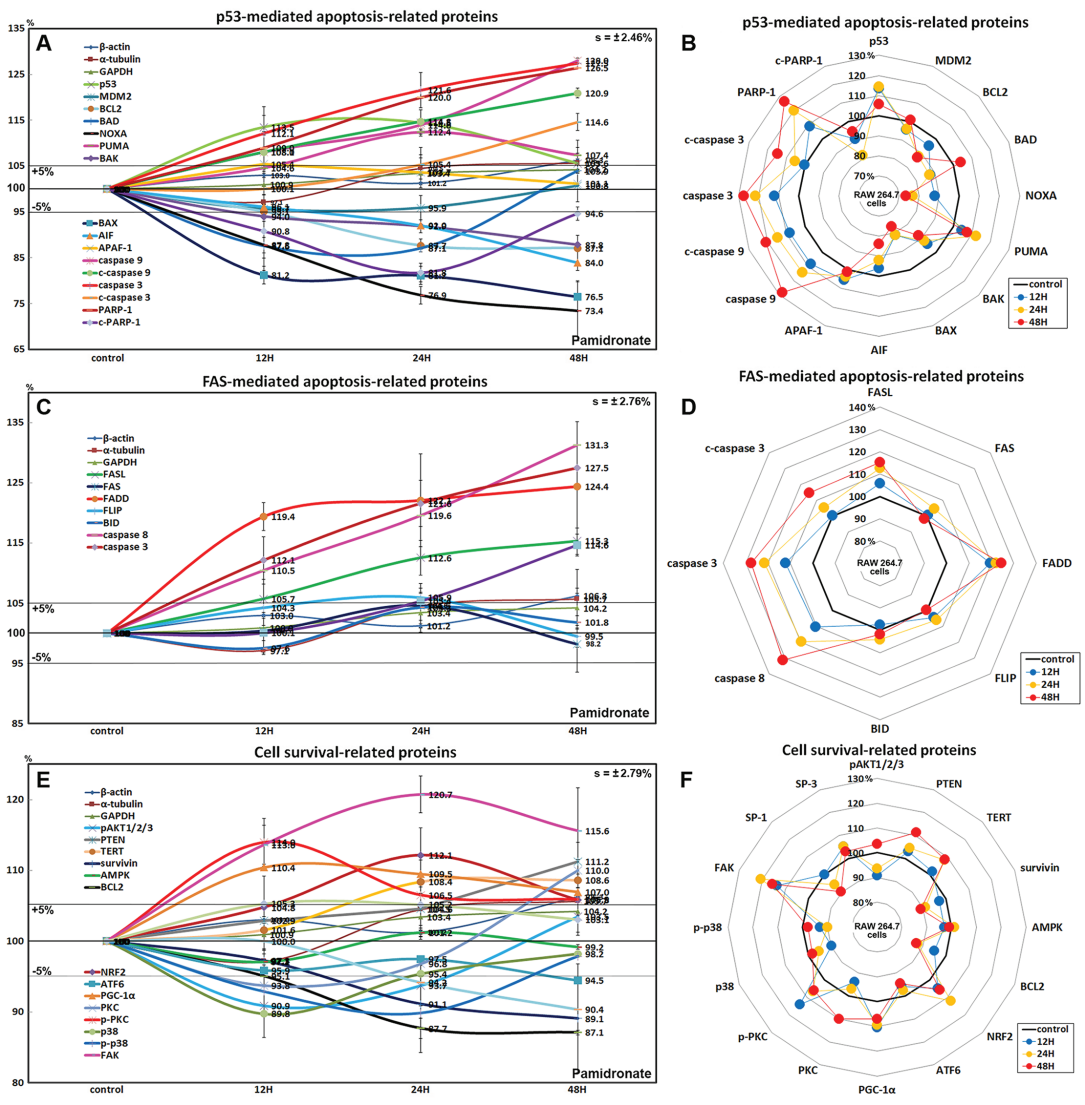
Expressions of p53-mediated apoptosis-related proteins, FAS-mediated apoptosis-related proteins, and cell survival-related proteins. Expressions of p53-mediated apoptosis-related proteins (A and B), FAS-mediated apoptosis-related proteins (C or D), and cell survival-related proteins (E and F) in RAW 264.7 cells treated with pamidronate for different times as determined by IP-HPLC. Line graphs (A), (C), and (E) show protein expressional changes on the same scale (%) vs. culture time (12, 24, or 48 h), whereas the star plots (B, D, and F) show the differential expression levels of proteins after 12, 24, or 48 h of treatment on appropriate scales (%). Standard error (s).

Pamidronate affected the expressions of p53-mediated apoptosis-related proteins, particularly p53 protein, which was increased by 14.5% after treatment for 24 h, while the expression of E3 ubiquitin-protein ligase MDM2 was decreased by 4.3% at 12 h vs. non-treated controls. After treatment for 48 h, the expressions of pro-apoptotic proteins, Bcl-2-associated death promoter (BAD), Bcl-2 homologous antagonist/killer (BAK), pro-apoptotic member of the Bcl-2 protein family NOXA, apoptosis regulator BAX, and apoptosis inducing factor (AIF) were decreased by 12.4%, 12.2%, 26.6%, 23.5%, and 16%, respectively, but the expressions of p53 upregulated modulator of apoptosis (PUMA) and apoptotic protease activating factor 1 (APAF-1) were increased by 12.4% and 5.4%. The expressions of apoptosis executor proteins, caspase 9, c-caspase 9, caspase 3, c-caspase 3, and poly [ADP-ribose] polymerase 1 (PARP-1) increased by 28%, 20.9%, 27.5%, 14.6%, and 26.5% at 48 h, whereas that of cleaved PARP-1 (c-PARP-1) was reduced by 18.2% at 24 h. On the other hand, the expression of the anti-apoptosis protein, BCL2 gradually decreased by 12.9% at 48 h (Figs. 5A and 5B). These results indicate pamidronate induced PARP-1/caspase 9/caspase 3-mediated apoptosis independently of p53/BAX and AIF signalings and in RAW 264.7 cells, which suggests pamidronate might induce PARP-1-mediated non-apoptotic cell death.

### Effects of pamidronate on the expressions of FAS-mediated apoptosis-related proteins in RAW 264.7 cells

RAW 264.7 cells treated with pamidronate showed increases in the expressions of FAS-mediated apoptosis-related proteins as compared with non-treated controls. After treatment with pamidronate for 48 h, the expressions of death receptors on cell surfaces, that is, of FAS, FAS ligand (FASL), and FAS-associated protein with death domain (FADD), were increased by 4.6%, 15.3%, and 24.4%, respectively, and those of caspase 8, caspase 3, and c-caspase 3 were also increased by 30.8%, 27.5%, and 14.6%, respectively. On the other hand, the expressions of FLICE-like inhibitory protein (FLIP) and BH3 interacting-domain death agonist (BID) were minimally changed (<±5%) (Figs. 5C and 5D). These findings indicate pamidronate might induce apoptosis via caspase 8 and 3 through FASL/FAS/FADD signaling in RAW 264.7 cells.

### Effects of pamidronate on the expressions of cell survival-related proteins in RAW 264.7 cells

RAW 264.7 cells treated with pamidronate showed variable changes in the expressions of cell survival-related proteins as compared with non-treated controls. The expressions of PTEN, telomerase reverse transcriptase (TERT), NRF2, PGC-1α, PKC, p-PKC, and focal adhesion kinase (FAK) were increased by 11.2%, 8.6%, 12.1%, 10.4%, 10%, 14%, and 13.7%, respectively, after 48 h of pamidronate treatment, while those of pAKT1/2/3, survivin, BCL2, p38, p-p38, and SP-1 were reduced by 9.1%, 10.9%, 12.9%, 10.2%, 10.2%, and 9.6%, respectively. On the other hand, the expressions of SP-3, AMPK, and ATF6 hardly changed (<±5%) (Figs. 5E and 5F). These results suggested cell survival was enhanced by the up-regulations of NRF2/PGC-1α and PKC/FAK signaling, which are features of mitochondrial biogenesis and the signal transduction cascade, respectively, but reduced by the down-regulations of AKT/survivin/BCL2 and p38/SP-1 signalings, which are features of cell exposure to stressors, such as oxidative damage. These results suggest that pamidronate increases energy metabolism and signal transduction in RAW 264.7 cells, but that the abilities of their cells to overcome different cytological stressors is relatively poor.

### Effects of pamidronate on the expressions of cell protection-related proteins in RAW 264.7 cells

The expressions of cell protection-related proteins in RAW 264.7 cells were increased by pamidronate; heat shock protein-70 (HSP-70) by 21.7% at 12 h, 1-phosphatidylinositol-4, 5-bisphosphate phosphodiesterase β-2 (PLC-β2) by 30.6% at 48 h, PI3K by 12.3% at 48 h, master regulator of mitochondrial biogenesis PGC-1α by 10.4% at 12 h, mitogen-activated protein kinase JNK-1 by 12.4% at 48 h, PKC by 10% at 48 h, p-PKC by 14% at 12 h, focal adhesion kinase (FAK) by 13.7% at 24 h, mucin 1 by 3.3% at 48 h, and mucin 4 by 10.8% at 24 h vs. non-treated controls, whereas the expressions of HSP-27, HSP-90, and 5’AMP-activated protein kinase (AMPK) were decreased by pamidronate; by 3.6% at 48 h, by 12.7% at 12 h, and by 2.9% at 12 h, respectively.

**Figure 6:**
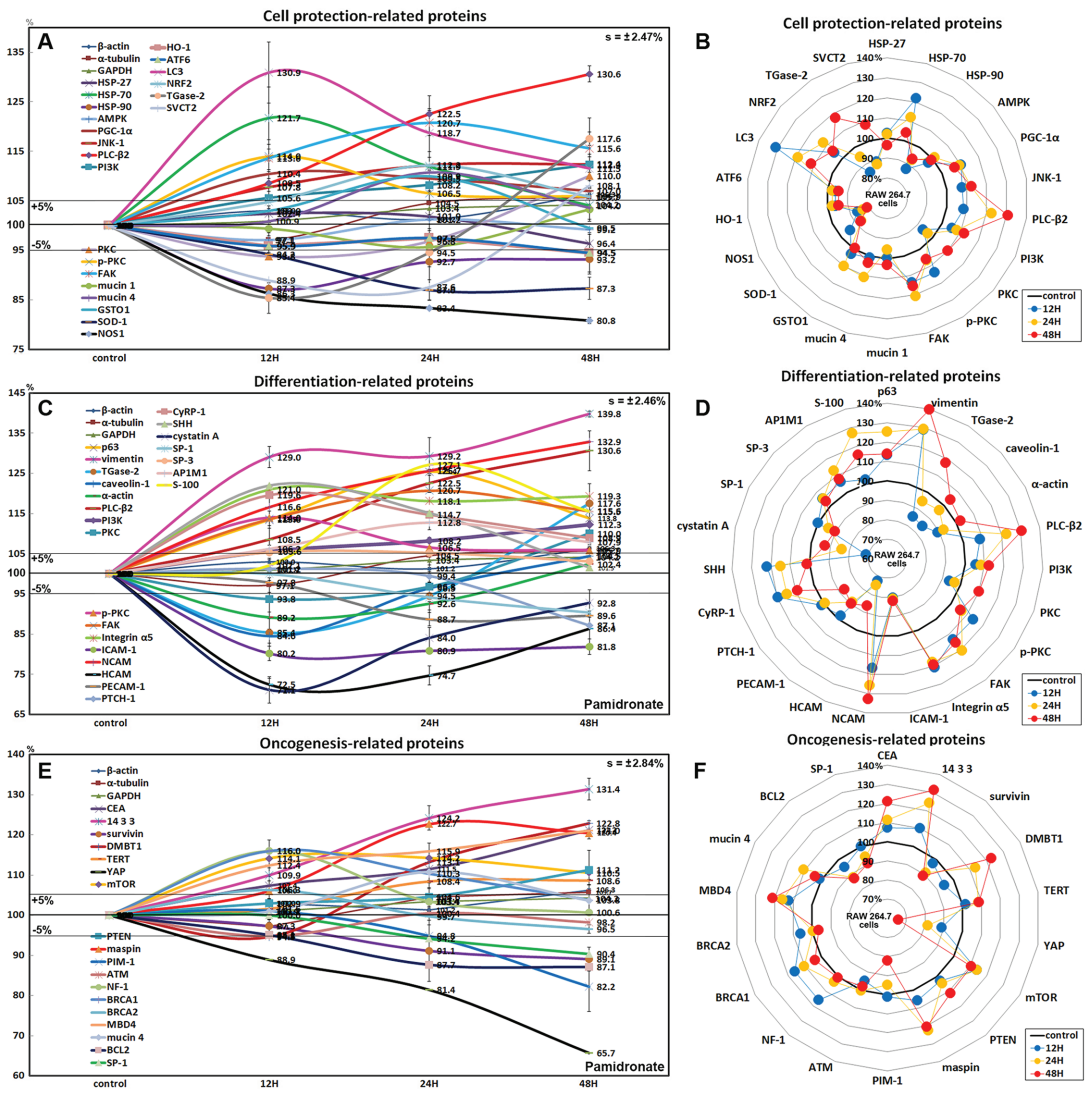
Expressions of cell protection-related proteins, differentiation-related proteins,and oncogenesis-related proteins. Expressions of cell protection-related proteins (A and B), differentiation-related proteins (C or D), and oncogenesis-related proteins (E and F) in pamidronate-treated RAW 264.7 cells as determined by IP-HPLC. Line graphs (A), (C) and (E) show protein expressional changes on the same scale (%) vs. culture time (12, 24, or 48 h), whereas the star plots (B, D and F) show the differential expression levels of proteins after 12, 24, or 48 h of treatment on appropriate scales (%). Standard error (s).

The expressions of anti-oxidative proteins in RAW 264.7 cells were increased by pamidronate; detoxifying enzyme glutathione S-transferase ω 1 (GSTO1) by 9.9% at 24 h, autophagy substrate LC3 by 30.9% at 12 h, NRF2 by 12.1% at 24 h, while the expressions of Cu-Zn superoxide dismutase-1 (SOD-1), nitric oxide synthase 1 (NOS1), heme oxygenase 1 (HO-1), and endoplasmic reticulum (ER) stress-regulated transmembrane transcription factor ATF6 were decreased by pamidronate; by 13% at 24 h, by 19.2% at 48 h, by 5.5% at 48 h, and by 5.5% at 48 h, respectively. And sodium-dependent vitamin C transporter 2 (SVCT2) and cross-linking enzyme transglutaminase 2 (TGase-2) were decreased by 11.1% and 14.6% at 12 h, respectively, but gradually increased by 8.1% and 17.6% at 48 h, respectively (Figs. 6A and 6B).

Although RAW 264.7 cells treated with pamidronate appeared to be silent, as they exhibited reduced NFkB signaling and had low levels of antioxidant-related proteins, SOD-1, NOS1, and HO-1 in their cytoplasms, they had higher levels of the cell protection-related proteins, HSP-70, PLC-β2, PI3K, PGC-1α, JNK-1, PKC, p-PKC, FAK, and mucin 1 and 4 than non-treated controls. These observations suggest the expressions of cellular protection-related proteins, such as those involved in detoxification and autophagy, are upregulated by pamidronate in RAW 264.7 cells despite reduced RAS and NFkB signalings.

### Effects of pamidronate on the expressions of differentiation-related proteins in RAW 264.7 cells

RAW 264.7 cells treated with pamidronate for 48 h showed increases in the expressions of the differentiation-related proteins p63 (25.4%), vimentin (39.8%), transglutaminase 2 (TGase-2, 17.6%), PLC-β2 (30.6%), PI3K (12.3%), PKC (10%), p-PKC (14%), FAK (20.7%), integrin α5 (21%), neural cell adhesion molecule (NCAM, CD56, 32.9%), cysteine rich protein-1 (CyRP-1, 19.6%), AP-1 complex subunit mu-1 (AP1M1, 12.8%), transcription factor SP-3 (5.3%), sonic hedgehog (SHH, 22%), and S-100 (27.1%) as compared with non-treated controls, but reductions in the expressions of the differentiation-related proteins, caveolin-1 (15.4%), α-actin (10.8%), intercellular adhesion molecule 1 (ICAM-1, CD54, 19.8%), homing cell adhesion molecule (HCAM, CD44, 27.5%), platelet endothelial cell adhesion molecule 1 (PECAM-1, CD31, 11.3%), receptor for sonic hedgehog PTCH-1 (12.9%), transcription factor SP-1 (9.6%), and cystatin A (28.9%) (Figs. 6C and 6D).

The proteins essential for the differentiation, migration, adhesion, and endocytosis of RAW 264.7 cells, that is, p63, vimentin, TGase-2, PLC-β2, PI3K, PKC, p-PKC, FAK, integrin α5, NCAM (CD56), CyRP-1, SP-3, SHH, and S-100 were upregulated by treatment with pamidronate for 48 h, whereas some proteins required for further differentiation into active macrophages or dendritic cells, that is, caveolin-1, α-actin, ICAM-1 (CD54), HCAM (CD44), PECAM-1 (CD31), PTCH-1, SP-1, and cystatin A, were downregulated. These observations suggest pamidronate-treated RAW 264.7 cells maintain major signal transduction organelles for cellular proliferation and protection but are defective in terms of advanced cytological differentiation due to reductions in the expressions of caveolin-1, α-actin, PTCH-1, and SP-1.

### Effects of pamidronate on the expressions of oncogenic proteins in RAW 264.7 cells

RAW 264.7 cells treated with pamidronate showed increases in the expressions of the oncogenic proteins, carcinoembryonic antigen (CEA, 21.2%), conserved regulatory molecule 14-3-3 (31.4%), deleted in malignant brain tumors 1 protein (DMBT1, 22.8%), telomerase reverse transcriptase (TERT, 8.6%), transmembrane subunit containing three EGF-like domains mucin 4 (10.8%), and serine protease inhibitor maspin (22.7%) as compared with non-treated controls, and also increases in the expressions of the tumor suppressor proteins, phosphatase and tensin homolog (PTEN, 11.2%), mTOR (14.2%), GTPase-activating protein neurofibromin 1 (NF-1, 16%), breast cancer type 1 susceptibility protein (BRCA 1, 16%), BRCA 2 (6.3%), and methyl-CpG binding protein-4 (MBD4, 21%). Whereas the expressions of strong oncogenic proteins, BCL2, SP-1, proto-oncogene serine/threonine-protein kinase PIM-1, and Yes-associated protein (YAP) were reduced by 12.9%, 9.6%, 17.4%, and 34.3%, respectively, after treatment for 48 h. In addition, the expression of tumor suppressor protein ATM was also diminished by 5.2% after treatment for 12 h (Figs. 6E and 6F).

Concomitant increases in the expressions of oncogenic proteins and tumor suppressor proteins indicate RAW 264.7 cells were stimulated by pamidronate and reacted by initiating oncogenic signaling for cellular proliferation, survival, and apoptosis.

### Effects of pamidronate on the expressions of angiogenesis-related proteins in RAW 264.7 cells

**Figure 7:**
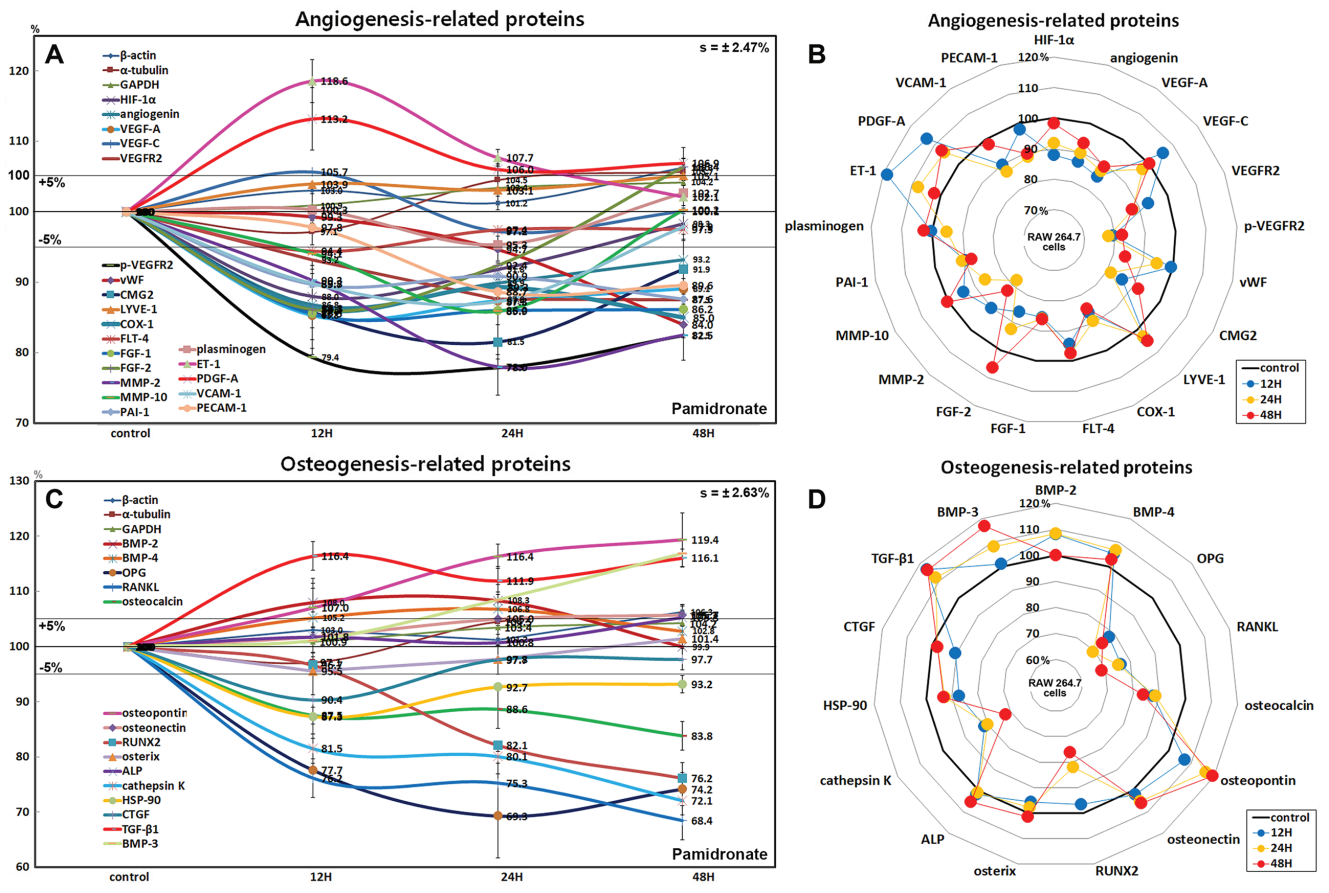
Expressions of angiogenesis-related proteins and of osteogenesis-related proteins. Expressions of angiogenesis-related proteins (A and B) and of osteogenesis-related proteins (C or D) in pamidronate-treated RAW 264.7 cells as determined by IP-HPLC. Line graphs (A) and (C) show protein expressional changes on the same scale (%) vs. culture time (12, 24, or 48 h), whereas the star plots (B and D) show the differential expression levels of proteins after 12, 24, or 48 h of treatment on appropriate scales (%). Standard error (s).

RAW 264.7 cells treated with pamidronate showed rapid reductions in the expressions of angiogenesis-related proteins, as follows, HIF-1α (12%), angiogenin (13.2%), vascular endothelial growth factor A (VEGF-A, 14.7%), VEGFR2 (12.5%), p-VEGFR2 (22.1%), von Willebrand factor (vWF, 16%), capillary morphogenesis protein 2 (CMG2, 18.5%), COX1 (11.6%), FGF-1 (14%), FGF-2 (13.9%), MMP-2 (22%), MMP-10 (14.1%), plasminogen activator inhibitor-1 (PAI-1, 12.4%), PECAM-1 (CD31, 11.3%), and vascular cell adhesion molecule-1 (VCAM-1, CD106, 12.7%) after treatment with pamidronate for 48 h vs. non-treated controls. The expressions of endothelin 1 (21-amino acid vasoconstricting peptide, ET-1) and PDGF-A were increased by 18.6% and 13.2%, respectively, whereas the expressions of VEGF-C, lymphatic vessel endothelial hyaluronan receptor 1 (LYVE-1), Fms-related tyrosine kinase 4 (FLT-4), and plasminogen barely changed (<±5%) (Figs. 7A and 7B).

Among the angiogenesis-related proteins, the expressions of the blood vessel-forming proteins, angiogenin, VEGF-A, VEGFR2, vWF, and CMG2 were markedly reduced by pamidronate, while those of the lymphatic vessel-forming proteins, VEGF-C and LYVE-1 tended to increase slightly (<5%). Pamidronate also reduced the expressions of the extracellular matrix proteins, FGF-1, FGF-2, MMP-2, and MMP-10, which are required for de novo angiogenesis and wound healing. These results suggest pamidronate significantly suppresses the expressions of angiogenesis-related proteins in RAW 264.7 cells, and that it might be able to potently inhibit blood vessel formation in vivo.

### Effects of pamidronate on the expressions of osteogenesis-related proteins in RAW 264.7 cells

Treatment with pamidronate for 48 h decreased the expressions of the osteogenesis-related proteins; osteoprotegerin (OPG, 30.7%), osterix (4.5%), mammalian Runt-related transcription factor 2 (RUNX2, 23.8%), osteocalcin (16.2%), and connective tissue growth factor (CTGF, 9.6%) and those of the osteoclastogenesis-related proteins; receptor activator of nuclear factor kappa-B ligand (RANKL, 31.6%), cathepsin K (27.9%), and HSP-90 (12.7%) vs. non-treated controls. On the other hand, the expressions of osteopontin and TGF-β1 were increased by pamidronate by 19.4% and 16.4% and the expressions of bone morphogenetic protein-2 (BMP-2, 8.3%), BMP-3 which negatively regulates bone density (16.8%), BMP-4 (6.8%), osteonectin (5.7%), and alkaline phosphatase (ALP, 5.3%), tended to be increased (Figs. 7C and 7D).

The expressions of the major osteoblast differentiation proteins; OPG, osteocalcin, and RUNX2, and of the osteoclast differentiation proteins; RANKL, HSP-90, and cathepsin K, were markedly reduced by 48 h of pamidronate treatment, whereas the expressions of the bone matrix proteins, osteopontin, BMP-2, BMP-4, osteonectin, and ALP tended to increase. In particular, the expressions of BMP-3 (an antagonist to other BMP’s in the differentiation of osteogenic progenitors) and TGF-β1 (an inhibitor of osteoclast activity) were markedly increased by pamidronate treatment. These results suggest pamidronate-treated RAW 264.7 cells are hardly differentiated into osteoclasts and give sparse influence on adjacent osteoblastic cells by expression of bone matrix proteins.

### Global protein expressions in pamidronate-induced RAW 264.7 cells

**Figure 8:**
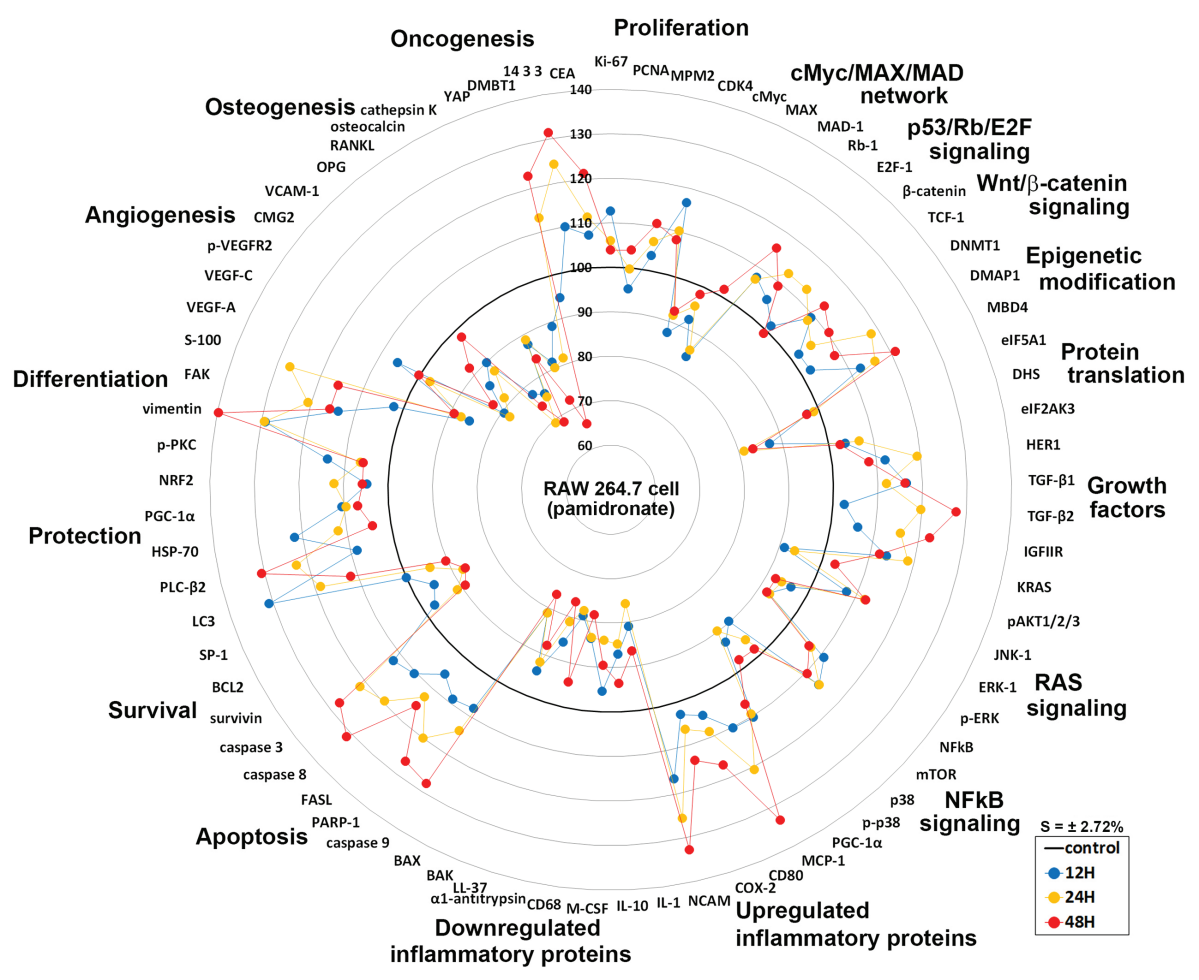
Star plot of global protein expression in pamidronate-treated RAW 264.7 cells. Star plot of global protein expression in pamidronate-treated RAW 264.7 cells. Representative proteins (*n* = 73) of each signaling pathway are plotted in a circular manner. The expressions of proliferation, some growth factors, cellular apoptosis, protection, and differentiation-related proteins were upregulated, while the expressions of protein translation-, cell survival-, angiogenesis-, and osteogenesis-related proteins were downregulated. RAS signaling and NFkB signaling were suppressed by the up-regulations of the downstream effector proteins, ERK-1 (p-ERK-1) and p38 (p-p38), respectively. The expressions of inflammatory proteins and oncogenesis-related proteins in RAW 264.7 cells were variably altered, but epigenetic methylation was increased by pamidronate treatment. Blue, yellow, and red spots indicate after 12, 24, and 48 h of pamidronate treatment, respectively.

Global protein expression changes of representative proteins (*n* = 73) from above 19 different protein signaling pathways are illustrated as a star plot in Fig. 8. Although pamidronate is low molecular weight entity, it was found to widely affect the expressions of proteins in different signaling pathways in RAW 264.7 cells. In particular, pamidronate inactivated epigenetic modification and protein translation and subsequently down-regulated the expressions of some proteins required for the proliferation, differentiation, protection, and survival of RAW 264.7 cells.

The increases observed in the expressions of proliferation-related proteins were presumably related to the up-regulations of p53/Rb/E2F and Wnt/β-catenin signaling by pamidronate albeit suppression of cMyc/MAX/MAD network signaling. The suppression of RAS signaling induced by pAKT1/2/3, ERK-1, and p-ERK-1 down-regulations was followed by cMyc/MAX/MAD network down-regulation and by a subsequent inhibition in RAW 264.7 cell proliferation. Furthermore, the marked suppression of NFkB signaling appeared to be associated with elevation of PARP-1- and FAS-mediated apoptosis and reduction of cellular differentiation, survival, immediate inflammatory reaction, and wound repair.

Overall changes in protein expressions induced by pamidronate affected the differentiation of RAW 264.7 cells and resulted in the productions of immature and/or inactive macrophages expressing lower levels of M2 macrophage differentiation proteins (wound healing proteins, TNFα, IL-1α, IL-6, IL-10, PECAM-1, CD99, VCAM, cathepsin G, cathepsin K, COX1, lysozyme, M-CSF, MMP-1, MMP-2, MMP-10, LL-37, α1-antitrypsin, β-defensin 1, β-defensin 2, and β-defensin 3), angiogenesis-related proteins (HIF-1α, angiogenin, VEGF-A, VEGFR2, p-VEGFR2, vWF, CMG2, COX1, FGF-1, FGF-2, MMP-2, MMP-10, PAI-1, PECAM-1, and VCAM-1), and osteoclast/osteoblast differentiation proteins (OPG, osterix, RUNX2, osteocalcin, and CTGF) and those of the osteoclastogenesis-related proteins (RANKL, cathepsin K, and HSP-90) than non-treated controls. Thus, pamidronate-treated RAW 264.7 cells simultaneously exhibited anti-inflammatory, anti-angiogenesis, and bone resorption inhibitive effects. However, the essential protein expression changes for cell proliferation, RAS signaling, and NFkB signaling rarely exceeded ±20%, which suggests pamidronate-treated cells exhibit relatively benign nature and be under homeostatic control.

### Highly up- and down-regulated proteins by pamidronate in RAW 264.7 cells

**Figure 9:**
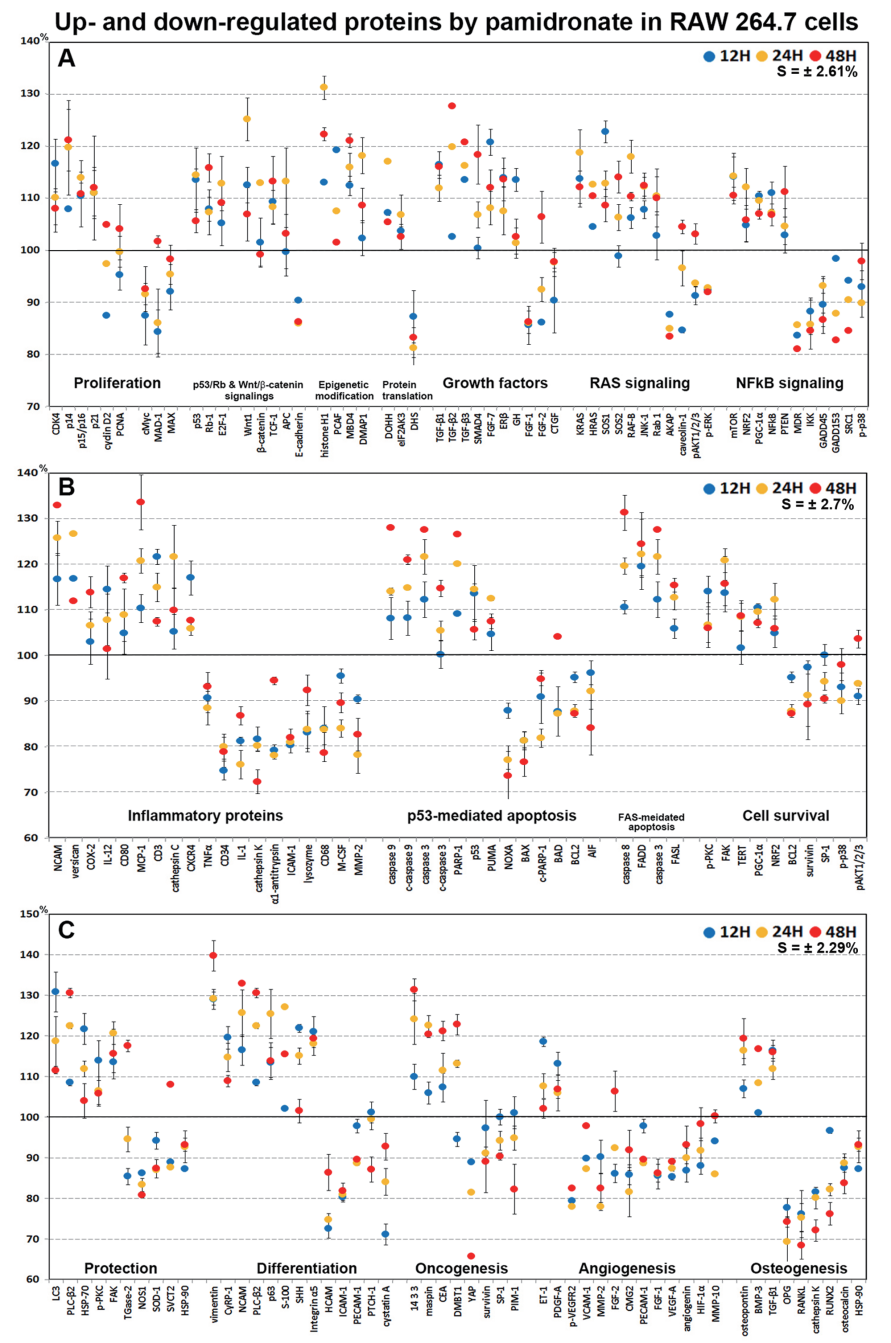
Highly up- and down-regulated proteins by pamidronate in RAW 264.7 cells. The cells were reactive to pamidronate by marked upregulation of some proteins for cellular proliferation, protection, differentiation, RAS signaling, NFkB signaling, and oncogenic proteins, but gradually degenerated by marked downregulation of M2 macrophage differentiation-related inflammatory proteins and survival-related proteins and by marked upregulation of apoptosis-related proteins. The major protein expressions for angiogenesis and osteoclastogenesis were dramatically suppressed (A–C). Blue, yellow and red spots indicate after 12, 24 and 48 h of pamidronate treatment, respectively.

In dot graphs plotted with highly up- and down-regulated proteins (>±10%, *n* = 155, Fig. 9), pamidronate-treated RAW 264.7 cells showed reactive upregulation (10–30%) of some proteins for cellular proliferation (CDK4, E2F-1, and TCF-1), protection (HSP-70, LC3, PLC-β2, and p-PKC), differentiation (vimentin, NCAM, p63, S-100, and SHH), RAS signaling proteins (KRAS, HRAS, SOS1, SOS2, RAF-B, JNK-1, and Rab), NFkB signaling proteins (NFkB, mTOR, NRF2, PGC-1α, and PTEN), and oncogenic proteins (DMBT1, 14-3-3, and CEA) vs. non-treated controls, and were tended to be proliferative but their cellular activities became abortive by the downregulation (10–20%) of some essential proteins (p14, p15/16, cMyc, MAX, MAD-1, E-cadherin, FGF-1, FGF-2, CTGF, AKAP, caveolin-1, MDR, IKK, GADD45, GADD153, SRC1, and p-p38) and by increases in the expression of histone and DNA methylation-related proteins (histone H1, MBD4, and DMAP1) and by decreases in the expressions of protein translation-related proteins (eIF2AK3 and DHS) (Figs. 9A and 9C).

On the other hand, pamidronate-treated RAW 264.7 cells appeared to be degenerated by marked downregulation (10–30%) of M2 macrophage differentiation-related inflammatory proteins (M-CSF, lysozyme, α1-antitrypsin, CD34, cathepsin K, and MMP-2) and survival-related proteins (BCL2, survivin, SP-1, and p-p38) and by marked upregulation (10–40%) of apoptosis-related proteins (caspase 9, c-caspase 9, caspase 3, c-caspase 3, PARP-1, p53, and PUMA) vs. non-treated controls. Subsequently, the major protein expressions for angiogenesis (VEGF-A, p-VEGFR2, angiogenin, HIF-1α, VCAM-1, FGF-1, FGF-2, PECAM-1, MMP-2, and MMP-10) and osteoclastogenesis (OPG, RANKL, cathepsin K, RUNX2, osteocalcin, and HSP-90) were dramatically suppressed (10–40%) by pamidronate (Figs. 9A–9C).

## Discussion

Pamidronate is a nitrogen-containing, synthetic bisphosphonate, and its phosphate groups are believed to interfere with phosphorylation processes or interact with proteins in cells (Chen et al., 2012; Nishida et al., 2003; Stefanucci, Marrone & Agamennone, 2015). Pamidronate is not sequestered as a waste material but relatively well adapted in cells, and thus, it is presumed pamidronate is maintained as a metabolite and influences not only the intracellular mevalonate pathway and protein isoprenylation but also signaling molecules and genetic materials (Henneman et al., 2011; Iguchi et al., 2010; Kaiser et al., 2013; Tatsuda et al., 2010). It has been shown pamidronate has considerable impact on cells such as macrophages, osteoclasts, and endothelial cells, and that its long-time usage is associated with the risk of BRONJ (Hoefert et al., 2015; Sharma et al., 2016; Zhang et al., 2013). In the present study, we assessed the effects of a therapeutic dose of pamidronate on the expressions of proteins in RAW 264.7 cells by IP-HPLC. As RAW 264.7 cells are derived from murine macrophages, and their immunological roles to dialyzed coffee extract were assessed by IP-HPLC (Yoon et al., 2018b), and this study also explored RAW 264.7 cells for their macrophage roles to pamidronate.

Pamidronate-induced proliferation of RAW 264.7 cells was examined by counting cell numbers directly on Petri dishes, and protein expressional changes were determined by IP-HPLC. The in situ proliferation index of pamidronate-treated RAW 264.7 cells over 24 h was 73.1 ± 2.32%, whereas that of non-treated cells was 69.9 ± 2.46%, thus the pamidronate-induced increase was 3.2%. Furthermore, this increase in in situ proliferation index matched the pamidronate-induced increases in the expressions of different proliferation-related proteins as determined by IP-HPLC. These data suggest pamidronate can slightly activate mitosis of murine macrophages, RAW 264.7 cells.

When we explored cellular mechanism responsible for altering protein expressions in RAW 264.7 cells, we noticed that the epigenetic environment was generally inactivated by pamidronate due to the up-regulations of DMNT1, MBD4, and DMAP1 and the down-regulation of KDM3D, which would tend to increase histone and DNA methylation levels. Protein translation was also inactivated by a marked reduction in DHS expression and an increase in eIF2AK3 (an inactivator of eIF2) expression vs. non-treated controls. We suggest the concurrent inactivations of epigenetic modification and protein translation by pamidronate may have reduced global RAW 264.7 cell activity.

Pamidronate-treated RAW 264.7 cells showed a marked reduction in cMyc/MAX/MAD network signaling during culture, and this was followed by the up-regulation of p27 (a negative regulator of G1 progression) by 16.7% at 48 h. Whereas p53/Rb/E2F signaling was enhanced by the up-regulations of p53, Rb-1, and CDK4 resulted in an increase in the expression of the objective transcription factor, E2F-1. Also, Wnt/β-catenin signaling was also enhanced by the up-regulations of Wnt-1, β-catenin, and snail, which led to the up-regulation of the objective transcription factor, TCF-1. As a result, the expressions of the proliferation-activating proteins Ki-67, PCNA, MPM2, CDK4, cyclin D2, and lamin A/C, were increased by pamidronate, and concurrently the expressions of the proliferation-inhibiting proteins p14, p15/16, p21, and p27 were compensatory increased during 48 h of pamidronate treatment. These results indicate pamidronate-treated RAW 264.7 cells were partly activated and proliferative due to increased p53/Rb/E2F and Wnt/β-catenin signaling despite a marked reduction in cMyc/MAX/MAD network signaling.

Pamidronate-treated RAW 264.7 cells showed increases in the expressions of some growth factors and associated proteins, such as IGF-1, IGFIIR, GH, HER1, HER2, TGF-β1, -β2, -β3, SMAD4, and ERβ, and subsequently, the expressions of upstream RAS signaling proteins including KRAS, HRAS, SOS-1, SOS-2, PI3K, JNK-1, and RAF-B were increased. However, downstreams of AKT and ERK signaling were reduced by the down-regulations of pAKT1/2/3, ERK-1, and p-ERK-1 and by the up-regulations of PTEN and mTOR. Consequently, RAS signaling was attenuated by the down-regulations of pAKT1/2/3, ERK-1, and p-ERK-1 in pamidronate-treated RAW 264.7 cells. Therefore, it appeared the pamidronate-induced negative regulation of RAS signaling might significantly reduce the expression of cMyc/MAX/MAD-1 network proteins.

Although the expressions of NFkB, NRF2, PGC-1α, PTEN, and mTOR tended to increase (<10%) after pamidronate treatment, the expressions of p38, p-p38, GADD45, GADD153, ATF6, MDR, and SRC-1 were reduced after 48 h of treatment. In addition, the expressions of the reactive oxygen and nitrogen species-related proteins SOD-1, NOS1, and HO-1 were consistently reduced by pamidronate. These observations indicate NFkB signaling was reduced due to pamidronate-induced suppression of the downstream effector protein p38 (p-p38) in RAW 264.7 cells, and that treated cells were less reactive to oxidative or endoplasmic reticulum stress than non-treated controls.

Although pamidronate suppressed RAS and NFkB signalings simultaneously, RAW 264.7 cells expressed higher levels of the protection-related proteins HSP-70, JNK-1, PLC-β2, LC3, and FAK, the cell survival-related proteins TERT, NRF2, PGC-1α, p-PKC, and FAK, and the oncogenesis-related proteins CEA, 14-3-3, and DMBT1 than non-treated controls. In particular, increases in the expressions of HSP-70 (protects against thermal and oxidative stress), JNK-1 (a mitogen-activated protein kinase responsible to different stress stimuli), LC3 (autophagosome biogenesis protein), NRF2 (transcription factor for many antioxidant genes), 14-3-3 (a regulator of diverse signaling proteins), DMBT1 (a glycoprotein containing multiple cysteine-rich domains that interact with tumor cells), and TERT (an RNA-dependent polymerase that lengthens telomeres in DNA strands) indicated pamidronate stressed RAW 264.7 cells and stimulated them to respond by expressing protection- and oncogenesis-related proteins.

Macrophages constitute a component of the front line of host defense and mediate innate immune responses by triggering; the productions of cytokines, chemokines, and cytotoxic molecules, the mobilizations of cells such as neutrophils and other leukocytes, the phagocytosis of pathogens and their delivery to lysosomes for degradation, and the induction of autophagy (Zhang et al., 2016). Many authors have reported macrophage functions are reduced after pamidronate treatment in vitro and in vivo (Escudero & Mandalunis, 2012; Hoefert et al., 2015; Hoefert et al., 2016a; Mian et al., 1994). In the present study, although the general cytodifferentiation proteins, p63, vimentin, PLC-β2, PI3K, PKC, FAK, integrin α5, SHH, and S-100 were upregulated by pamidronate, the M2 macrophage differentiation-related proteins, TNFα, lysozyme, cathepsin G, cathepsin K, M-CSF, ICAM-1, and α1-antitrypsin were consistently downregulated, which suggested pamidronate prevented the differentiation of RAW 264.7 cells into active M2 macrophages, and resulted retarded wound healing after pamidronate treatment in vivo (Ariza Jimenez et al., 2018; Chen, Cheng & Feng, 2018).

Pamidronate-treated RAW 264.7 cells also showed increases in the expressions of the apoptosis executor proteins, caspase 8, caspase 3, and c-caspase 3, which are activated by the FAS-mediated apoptosis signaling cascade, and that the expressions of caspase 9 and c-caspase 9 were also increased by p53 upregulated modulator of apoptosis (PUMA) and APAF-1 even though the expressions of the upstream p53-mediated apoptosis signaling proteins, BAD, BAK, BAX, NOXA, and BCL2 were suppressed. In addition, the expression of PARP-1 was increased by pamidronate whereas the expression of cleaved PARP-1 (c-PARP-1) was decreased. These results suggest pamidronate-treated RAW 264.7 cells underwent FAS/caspase 3/PARP-1-mediated apoptosis, that is, parthanatos, due to the accumulation of polymeric adenosine diphosphate ribose (poly (ADP-ribose) or PAR) caused by severe DNA damage. Actually, pamidronate-treated RAW 264.7 cells were continuously proliferative as evidenced by the up-regulations of p53/Rb/E2F and Wnt/β-catenin signaling, though they only showed a slight increase in cell numbers after 24 h of pamidronate treatment vs. non-treated controls, which suggests some cells unable to differentiate into mature macrophages may have succumbed to FAS-mediated or PARP-1-associated apoptosis.

Pamidronate reduced the expressions of the osteoclastogenesis-related proteins, RANKL and cathepsin K in RAW 264.7 cells, indicating it inhibited osteoclast differentiation, which is in-line with the reported disappearance of osteoclasts in bisphosphonate-treated animals (Kameka et al., 2014; Kawata et al., 2004; Mayahara & Sasaki, 2003) and has implications regarding the effects of pamidronate effects on osteolytic diseases such as including osteoporosis, fibrous dysplasia, Paget’s disease, and Gorham’s disease (Hammer et al., 2005; Kravets, 2018; Saraff et al., 2018), etc.

Pamidronate also downregulated the osteoblast differentiation proteins OPG, RUNX2, osterix, and osteocalcin but slightly induced the expressions of bone matrix proteins such as osteopontin, BMP-2, BMP-4, osteonectin, and ALP together with BMP-3 which negatively regulates bone density. These findings may be relevant to the osteoinductive effects of low-dose bisphosphonate reported in chronic periodontitis and after dental implantation (Alqhtani et al., 2017; Ata-Ali et al., 2016; Bhavsar et al., 2016; Khojasteh, Dehghan & Nazeman, 2019). However, pamidronate-treated RAW 264.7 cells may negatively regulate cytodifferentiation to osteoblasts in vivo and their abnormal bone production can contribute to the disruption of Haversian system canaliculi, which leads osteocyte death and increases the risk of osteonecrotic infections like BRONJ (Acevedo et al., 2015; Favia, Pilolli & Maiorano, 2009; Park et al., 2009).

Interestingly, pamidronate altered expressions of inflammatory proteins in RAW 264.7 cells both positively and negatively. The expressions of inflammatory proteins that participate in immediate inflammatory reaction, for example, TNFα, IL-1, lysozyme, CD68, LL-37, and β-defensin-1, -2, -3, were markedly reduced, whereas those that participate in delayed inflammatory reaction, for example, CD3, CD80, Pdcd-1/1, IL-12, and MCP-1, were elevated. The inhibition of immediate inflammatory reaction results the failure of innate immunity, and is relevant to severe necrotic infection of BRONJ involved with reduction of granulation tissue (Burr & Allen, 2009; Carmagnola et al., 2013; Marx & Tursun, 2012; Ziebart et al., 2011). Actually, pamidronate markedly suppressed the expressions of the angiogenesis-related proteins, HIF-1α, VEGF-A, VERFR2, p-VEGFR2, vWF, CMG2, FGF-1, FGF-2, MMP-2, MMP-10, COX-1, PAI-1, VCAM-1, and PECAM-1 in RAW 264.7 cells vs. non-treated controls but had relatively little effect on the expressions of the lymphatic vessel-related proteins, VEGF-C, LYVE-1, and FLT-4. These observations suggest that pamidronate-treated RAW 264.7 cells do not participate in immediate inflammatory reactions and vascular capillary production, but that they still provide some support for lymphatic drainage.

Pamidronate was found to widely affect the expressions of proteins in different signaling pathways in RAW 264.7 cells. Its global protein expression changes were illustrated in Fig. 8, exhibiting dynamic impacts on epigenetic modification, protein translation, RAS signaling, NFkB signaling, cellular proliferation, protection, differentiation, survival, apoptosis, inflammation, angiogenesis, and osteoclastogenesis. Highly up- and down-regulated proteins for each cellular functions were summarized in Fig. 9. Pamidronate induced marked over- and under-expression of some elective proteins more than 20% compared to non-treated controls, which may play pathogenetic roles (biomarkers) for cellular differentiation, inflammation, apoptosis, angiogenesis, and osteoclastogenesis in RAW 254.7 cells.

## Conclusions

Summarizing, pamidronate was found to alter the expressions of many important proteins in RAW 264.7 cells. It upregulated proliferation-related proteins associated with p53/Rb/E2F and Wnt/β-catenin signaling and inactivated epigenetic modification and protein translation. In addition, RAS (cellular growth) and NFkB (cellular stress) signalings were markedly affected by pamidronate. Pamidronate-treated cells showed that upstream of RAS signaling was stimulated by up-regulation of some growth factors, while downstream of RAS signaling was attenuated by down-regulation of ERK-1 and p-ERK-1, resulted in reduction of cMyc/MAX/MAD network expression. They also showed suppression of NFkB signaling by downregulating p38 and p-p38 and upregulating mTOR. Consequently, pamidronate affects global protein expression in RAW 264.7 cells by downregulating the expressions of immediate inflammation, cellular differentiation, survival, angiogenesis, and osteoclastogenesis-related proteins, but by upregulating PARP-1- and FAS-mediated apoptosis, protection, and proliferation-related proteins. These findings suggest pamidronate has potent anti-inflammatory, anti-angiogenesis, and anti-osteoporotic effects together with cellular stresses dysregulating RAS signaling, NFkB signaling, apoptosis, and proliferation. The present study explored the global expressions of representative essential proteins (*n* = 218) in pamidronate-treated RAW 264.7 cells, but some affected proteins were so dynamic and variable that they should be continuously monitored by IP-HPLC, if pamidronate treatment will be prolonged. Finally, we suggest further molecular biologic studies be undertaken on interactions between pamidronate and target proteins.

## Supplemental Information

10.7717/peerj.9202/supp-1 Supplemental Information 1 Mathematical algorithm for IP-HPLC analysis. From this algorithm, the relative ratio (%) between objective protein level and control protein level can be obtained, albeit it is impossible to get the concentration of objective protein through IP-HPLC.

10.7717/peerj.9202/supp-2 Supplemental Information 2 IP-HPLC experimental data of pamidronate-induced protein expressions in RAW 264.7 cells. Analysis of IP-HPLC.

10.7717/peerj.9202/supp-3 Supplemental Information 3 Representative chromatography through IP-HPLC analysis. Analysis of IP-HPLC.

## Additional Information and Declarations

### Data Availability

The following information was supplied regarding data availability: The raw measurements and raw data are available in the Supplemental Files.
