# Supplementary material for: Extensive protein expression changes induced by pamidronate in RAW 264.7 cells as determined by IP-HPLC
Source: PeerJ. 2020 May 21;8:e9202. doi: 10.7717/peerj.9202 (PMC7246033; doi:10.7717/peerj.9202)
Supplement: Supplemental Information 3 — Analysis of IP-HPLC. [file peerj-08-9202-s003.docx]

**Supplementary data 2**

**Representative chromatography through IP-HPLC analysis**


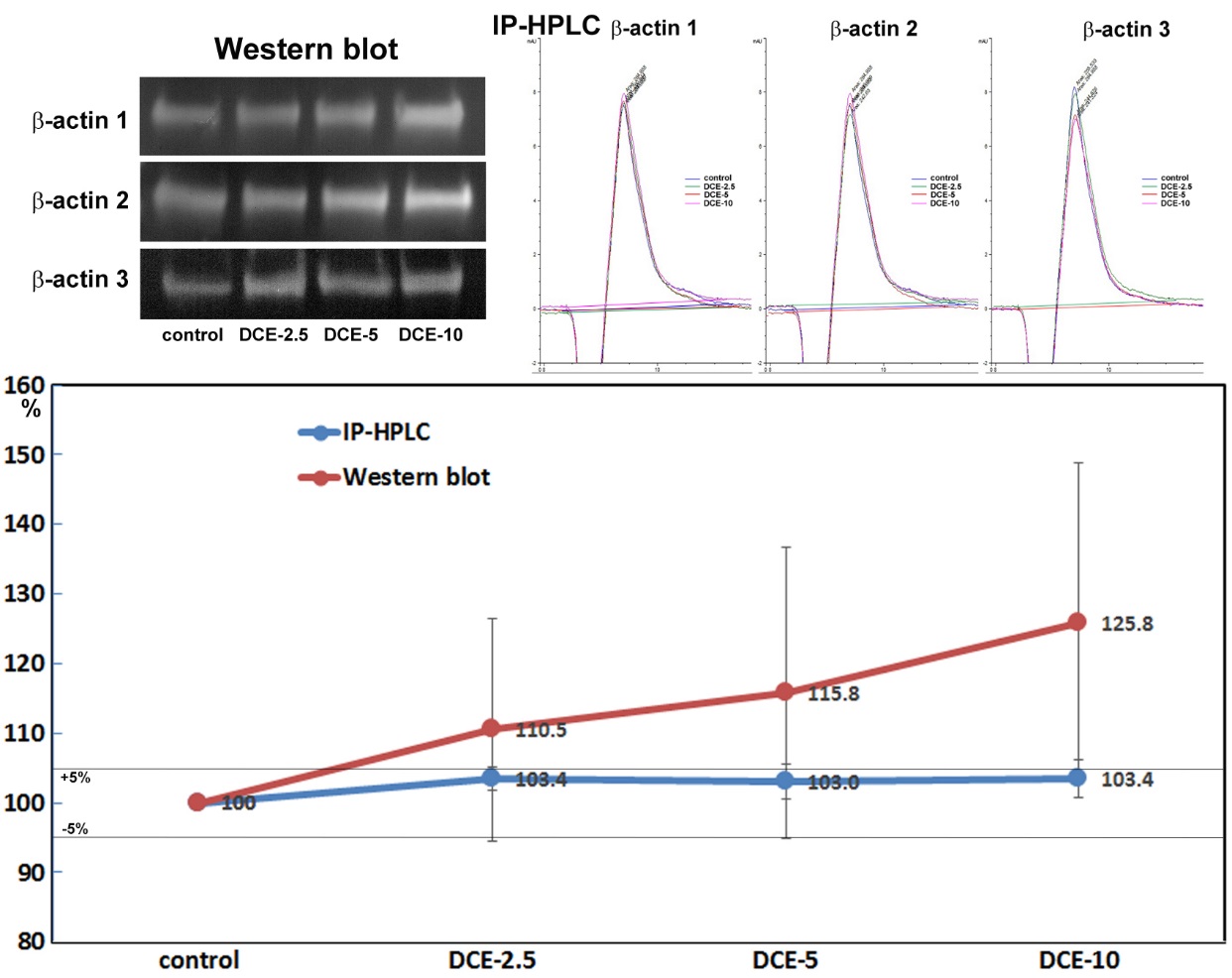


**Supplementary Figure 2.** β-Actin expression in DCE-treated RAW 264.7 cells was explored through western blot and IP-HPLC. Densitometry data of triplicated western blot (red line) showed big standard deviation (16.1 – 23.2 %), while triplicated IP-HPLC data (blue line) showed relatively small standard deviation (1.7 – 2.7%). Therefore, the latter was available to perform statistical analysis contrary to the former. These data were obtained from the previous study (Yoon, C.S., Kim, M.K., Kim, Y.S. & Lee, S.K. *In vitro* protein expression changes in RAW 264.7 cells and HUVECs treated with dialyzed coffee extract by immunoprecipitation high performance liquid chromatography. *Scientific reports* **8**, 13841 (2018))

*
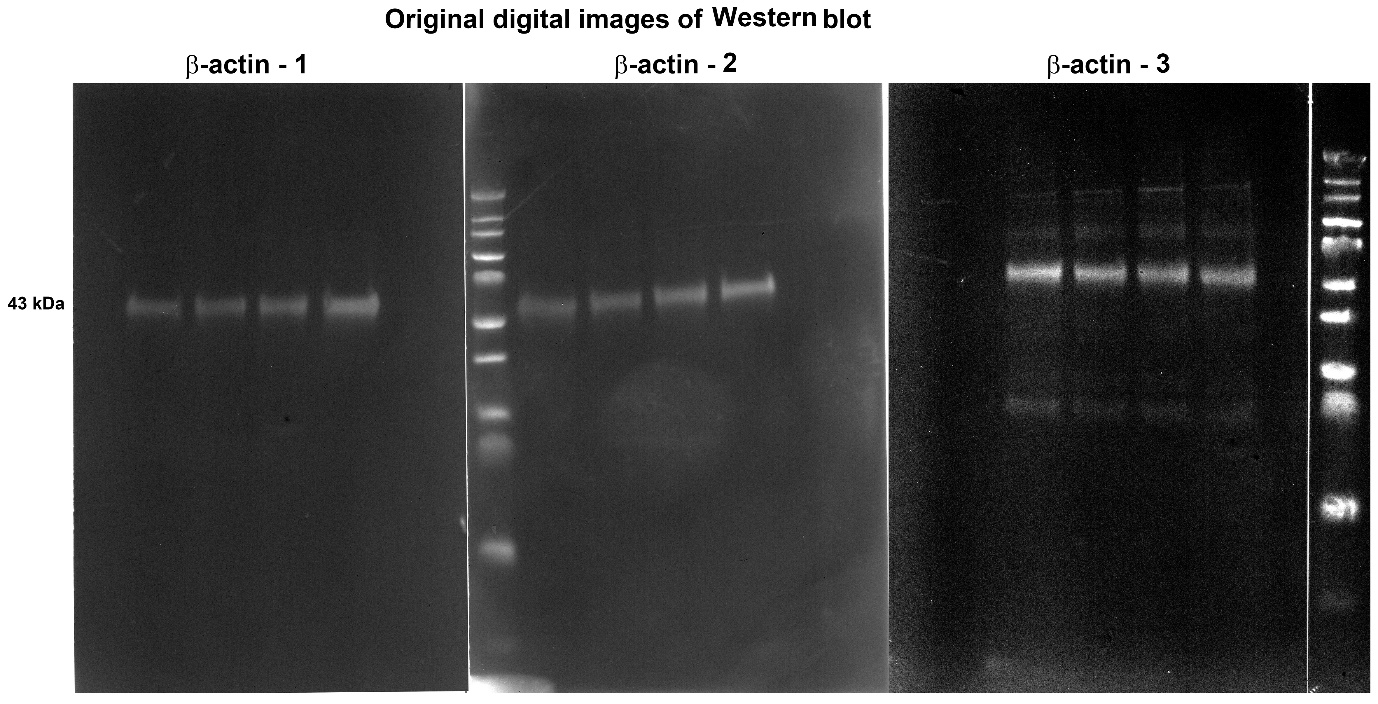
*
